# Maternal Preconception Antibiotic Exposure Disrupts Microbial Succession: A Transgenerational Risk for Offspring Gut Mucosal Immaturity and Colitis Susceptibility

**DOI:** 10.1002/advs.202516931

**Published:** 2026-04-02

**Authors:** Yuzhu Chen, Ruqiao Duan, Cunzheng Zhang, Gaonan Li, XiaoLin Ji, Qi Zhang, Fei Pei, Kun Wang, Liping Duan

**Affiliations:** ^1^ Department of Gastroenterology Peking University Third Hospital Beijing China; ^2^ PKUMed‐Wisbiom Joint Laboratory for Human Microbiome Research Beijing China; ^3^ Department of Gastroenterology, The Affiliated Suzhou Hospital of Nanjing Medical University, Suzhou Municipal Hospital, Gusu School Nanjing Medical University Jiangsu China; ^4^ Department of Pathology Peking University Third Hospital Beijing China

**Keywords:** antibiotics, gut microbiome, inflammatory bowel disease, offspring health, preconception

## Abstract

The early‐life microbiome plays a pivotal role in host development and lifelong health. Maternal factors are increasingly recognized as crucial in shaping offspring microbiome. However, how maternal preconception perturbations affects offspring health remain unclear. Thus, we combined animal and clinical data to elucidate whether preconception microbial perturbations disrupt microbial succession and increase offspring susceptibility to colitis. In animals, preconception antibiotic exposure induced long‐lasting disruptions in offspring microbial ecology, through enhanced maternal‐offspring microbial transmission, altered microbial developmental trajectories, and increased selective pressures during microbial community assembly. Ultimately, these alterations resulted in persistent gut mucosal immaturity and heightened susceptibility to colitis in adulthood. Complementary clinical studies revealed concordant alterations in gut microbiome and metabolome of children with inflammatory bowel disease (IBD) and their seemingly healthy mothers, characterized by pro‐inflammatory taxa and metabolites. Notably, mothers of IBD children reported significantly higher antibiotic exposure than controls, which was also associated with enhanced maternal‐offspring microbial transmission and increased selective pressures during microbial community assembly. Our findings reveal a potential intergenerational mechanism in which preconception perturbations are associated with disrupted microbial succession, transgenerational propagation of gut mucosal immaturity, and susceptibility to colitis. These results underscore the importance of judicious antibiotic use during the often‐overlooked preconception period.

## Graphical abstract

This study reveals that maternal antibiotic exposure prior to conception disrupts intergenerational gut microbial succession. By enhancing maternal‐offspring microbial transmission, altering microbial developmental trajectories and increasing selective pressures during community assembly, these disturbances lead to persistent gut mucosal immaturity and an elevated susceptibility to colitis in adulthood. These findings underscore the importance of judicious antibiotic use during the often‐overlooked preconception period.

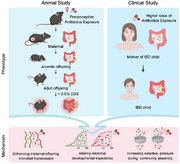

## 1 Introduction

The early‐life microbiome lays the groundwork for host immunological, metabolic, and physiological development, with far‐reaching implications for lifelong health [1, 2, 3]. Although postnatal factors, such as diet and environment influence microbiome formation, maternal factors are increasingly recognized as key determinants of microbial colonization across generations [4, 5, 6]. Notably, the maternal microbiome serves as the primary source of microbial inoculum for offspring through vertical transmission, shaping the initial trajectory of microbial succession. Although changes in the maternal gut microbiome during pregnancy are known to impair offspring health [7, 8, 9, 10], the long‐term effects of preconception microbiome perturbations remain largely uncharted.

Inflammatory bowel disease (IBD) is closely associated with disruptions in the early‐life microbiome. The rising global incidence of pediatric IBD (PIBD) highlights the urgent need to identify preventable risk factors [11, 12, 13]. Evidence suggests that IBD pathogenesis begins years before diagnosis, with early‐life exposures, including maternal factors, potentially setting the stage for latent disease [14, 15]. Epidemiological studies also link perinatal antibiotic use, delivery mode, and feeding practices to altered offspring microbiome composition and increased IBD risk [16, 17, 18]. However, whether maternal microbial perturbations specifically induced before conception are associated with altered foundational microbial succession and propagate gut pathology across generations remains unclear.

Although most focus on pregnancy, the role of the preconception microbiome as an intergenerational catalyst is often overlooked, despite evidence that antibiotic‐induced microbial alterations can persist for months [19]. Current studies link maternal microbial perturbations to offspring microbiome shifts or disease susceptibility but do not explain how these changes disrupt microbial assembly programs to directly cause structural gut pathology, a potential precursor to IBD [20].

To address these gaps, we combined controlled animal models with clinical cohort studies and applied microbiome–metabolome multi‐omics profiling. This integrated approach enabled us to investigate how maternal preconception microbial perturbations shape offspring gut development, microbial maturation, and susceptibility to colitis, as well as to uncover potential mechanisms. Notably, by linking coordinated mother–child microbiome shifts to maternal antibiotic exposure, we demonstrate the relevance of these findings to human IBD. This study highlights the preconception phase as a critical window during which antibiotic stewardship and microbiota monitoring could help prevent offspring susceptibility to colitis.

## 2 Results

### 2.1 Preconception Antibiotic Exposure Induces Lasting Intestinal Damage and Colitis Susceptibility in Offspring

**FIGURE 1:**
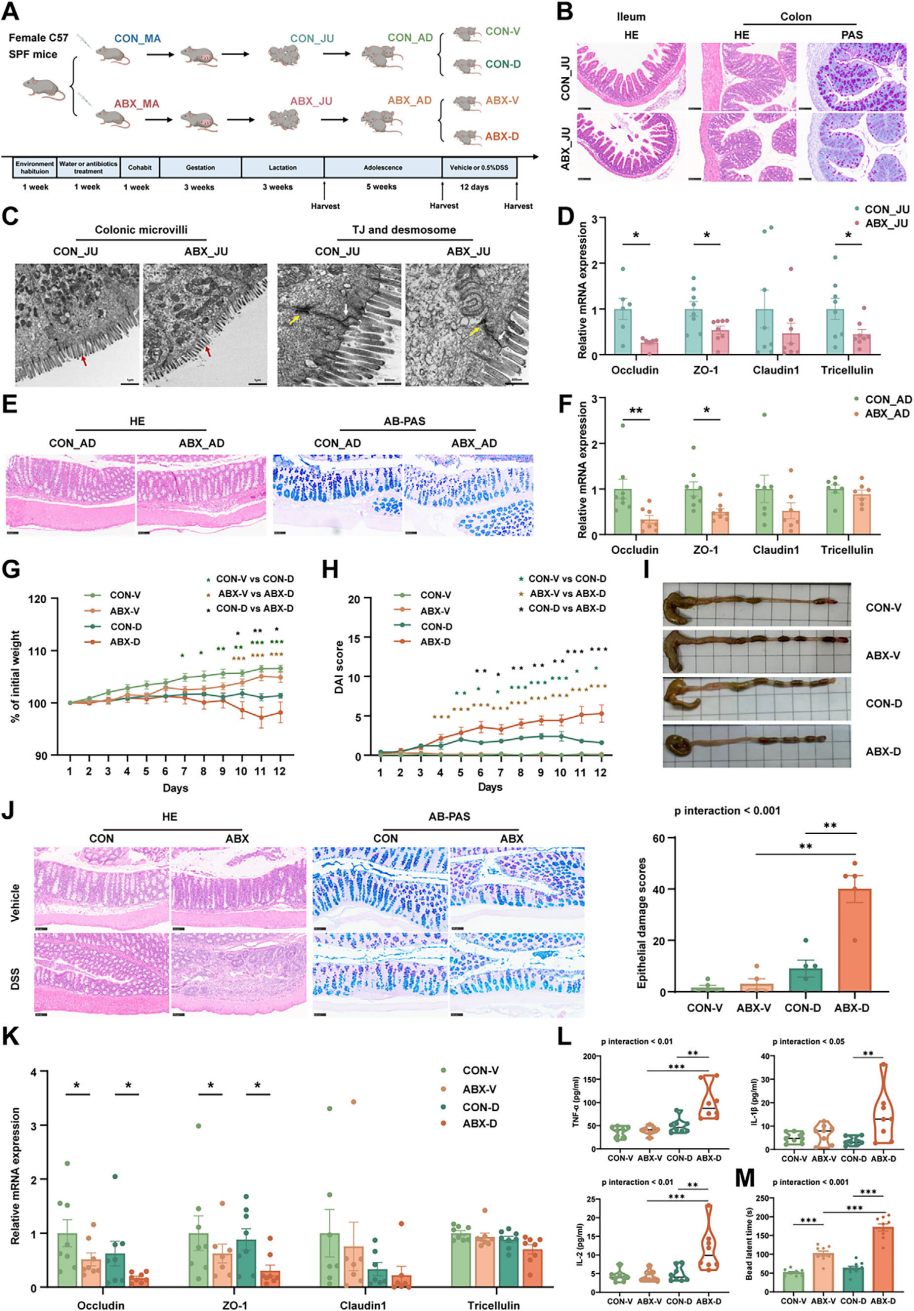
Maternal preconception antibiotic exposure leads to intestinal mucosal immaturity in offspring and increased colitis susceptibility in adulthood. (A) Schematic of experimental design: Female mice were orally gavaged with sterile water (CON) or antibiotics (ABX) for 1 week, followed by mating with male mice. Offspring were collected at 3 (both male and female) and 8 weeks of age for analysis. The 8‐week‐old offspring were administered either sterile water or a 0.5% DSS solution for 12 consecutive days. (B) Representative images of hematoxylin and eosin (H&E) staining of the ileum, and H&E and Periodic‐acid Schiff (PAS) staining of the colon (scale bar = 100 µm). (C) Representative images of the colon ultrastructure in juvenile offspring under transmission electron microscopy. Red, white, and yellow arrows indicate microvilli, tight junctions between epithelial cells, and desmosomes, respectively (scale bar = 1 µm or 500 nm). (D) Relative mRNA expression of colonic barrier‐related genes (Occludin, ZO‐1, Claudin1, and Tricellulin) in juvenile offspring (*n* = 6‒8). (E) Representative images of H&E and Alcian Blue‐Periodic Acid Schiff (AB‐PAS) staining of the colon (scale bar = 100 µm). (F) Relative mRNA expression of colonic barrier‐related genes (Occludin, ZO‐1, Claudin1, and Tricellulin) in adult offspring (*n* = 7‒8). (G) Normalized percentage weight loss of adult offspring during administration of sterile water or 0.5% DSS solution (*n* = 8). (H) Disease activity index (DAI) scoring of adult offspring during administration of sterile water or 0.5% DSS solution (*n* = 8). (I) Representative colon images of adult offspring after administration of sterile water or 0.5% DSS solution. (J) Representative images of H&E and AB‐PAS staining of the colon (scale bar = 100 µm). Histopathological scores after administration of sterile water or 0.5% DSS solution (*n* = 5). (K) Relative mRNA expression of colonic barrier‐related genes (Occludin, ZO‐1, Claudin1, and Tricellulin; *n* = 7‒8). (L) Concentrations of inflammatory factors (TNF‐α, IL‐1β, and IL‐2; *n* = 8). (M) Colonic transport time (*n* = 8). Data are presented as mean ± SEM. Mann‒Whitney *U*‐test was used for comparisons between two groups (D,F). Two‐way repeated measures ANOVA followed by Sidak's multiple comparison test was used (G, H). Two‐way ANOVA followed by Tukey's (J, L, M) or Sidak's (K) multiple comparison test was used for analyses involving two variables. *p < 0.05, **p < 0.01, ***p < 0.001.

To explore the intergenerational effects of maternal preconception antibiotic exposure on offspring intestinal development and colitis susceptibility, we established a murine model. Female mice were administered antibiotics (ABX) or vehicle (CON) for 1 week before mating (Figure 1A). Given the critical role of lactation in mammalian growth and development, we first examined juvenile offspring (JU) of both sexes at weaning (3 weeks old). Offspring from the ABX group exhibited ileal villus shortening, reduced colonic crypt depth, and diminished goblet cell numbers (Figure 1B and Figure S1A). Transmission electron microscopy revealed shortened and loosened colonic microvilli, abbreviated tight junctions (TJs), and desmosome fragmentation (Figure 1C and Figure S1B). A significant downregulation of key TJ genes (Occludin, ZO‐1, and Tricellulin) compared with that in controls was observed (Figure 1D). Further, no differences were observed between males and females in intestinal mucosal immaturity (Figure S1C,D). Next, we investigated whether the intestinal mucosal immaturity observed in juvenile offspring persisted into adulthood (AD) (8 weeks old). Reduced colonic crypt depth and goblet cell depletion were evident in the ABX_AD group (Figure 1E and Figure S1E). Occludin and ZO‐1 expression remained suppressed, whereas Tricellulin levels normalized (Figure 1F). No significant differences were observed in the colonic cytokine levels (Figure S1F).

Given the baseline intestinal mucosal immaturity, we challenged adult offspring with 0.5% dextran sulfate sodium (DSS; CON‐D and ABX‐D) to assess colitis susceptibility, with vehicle‐treated groups (CON‐V and ABX‐V) serving as controls (Figure 1A). Compared with CON‐D offspring, ABX‐D offspring displayed exacerbated weight loss, elevated disease activity index (DAI), and pronounced colon shortening (Figure 1G‒I and Figure S1G). Histologically, ABX‐D offspring showed severe epithelial erosion and further reduction in goblet cell numbers (Figure 1J and Figure S1H), whereas colonic crypt depth was not significantly different (Figure S1H). TJ genes, Occludin, and ZO‐1, were downregulated post‐DSS treatment (Figure 1K). Moreover, pro‐inflammatory cytokines, including TNF‐α, IL‐1β, and IL‐2, increased significantly (Figure 1L and Figure S1I). ABX‐D offspring exhibited slower colonic transit than ABX‐V and CON‐D offspring, whereas small intestinal transit remained unaffected (Figure 1M and Figure S1J).

These findings indicate that maternal preconception antibiotic exposure induces persistent intestinal mucosal immaturity and barrier dysfunction, persisting from juvenile to adulthood, thereby conferring markedly heightened susceptibility to colitis.

### 2.2 Preconception Antibiotic Exposure Triggers Persistent Intergenerational Microbial Perturbations and Conserved Microbial‒Metabolic Signatures

To assess whether preconception antibiotic‐induced maternal microbiota alterations permanently reshaped offspring microbial ecology, we performed metagenomic sequencing and untargeted metabolomics analyses at three key stages in mouse model: late gestation dams (MA), 3‐week‐old JU, and 8‐week‐old AD. This approach allowed us to systematically compare microbial taxonomy, function, and metabolic profiles between maternal antibiotic‐exposed (ABX) and control (CON) groups.

**FIGURE 2:**
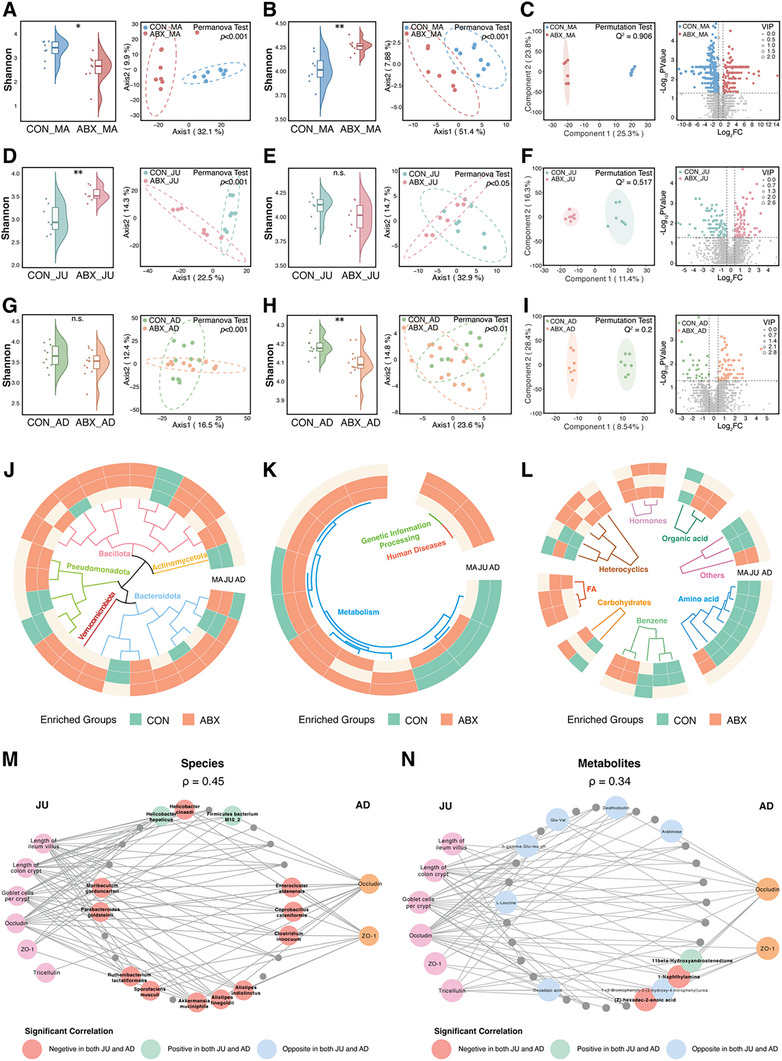
Maternal preconception antibiotic exposure persistently alters gut microbial and metabolic profiles and establishes intergenerationally conserved host–microbe–metabolite interactions. (A,B) α diversity (Shannon index) and β diversity (PCoA based on Aitchison distance) of maternal gut microbiota (*n* = 8) at the species level (A) and KEGG Level 3 functional pathways (B). (C) OPLS‐DA and volcano plot of maternal gut metabolites (*n* = 6). (D,E) Shannon index and PCoA on Aitchison distance of juvenile offspring gut microbiota (*n* = 7) at the species level (D) and KEGG Level 3 pathways (E). (F) OPLS‐DA and volcano plot of juvenile offspring gut metabolites (*n* = 7). (G,H) Shannon index and PCoA of Aitchison distance of adult offspring gut microbiota (*n* = 10‒12) at the species level (G) and KEGG Level 3 pathways (H). (I) OPLS‐DA and volcano plot of adult offspring gut metabolites (*n* = 7). (J) Circular heatmap of intergenerationally consistent differential species annotated using a phylogenetic tree. (K) Circular heatmap of intergenerationally consistent differential functional pathways (KEGG Level 3) annotated using hierarchical functional classification. (L) Circular heatmap of intergenerationally consistent differential metabolites annotated using metabolic class information. (M,N) Correlation networks between conserved species in (M), metabolites in (N), and host phenotypes in juvenile (JU) and adult (AD) offspring. In the network, edges represent significant Spearman correlations with host phenotypes (*p* < 0.05, |R| > 0.5). Red nodes indicate features negatively correlated with juvenile and adult phenotypes, green nodes indicate features positively correlated with both stages, blue nodes indicate discordant correlations between stages, and gray nodes indicate non‐significant correlations. α diversity was assessed using the Wilcoxon rank‐sum test. Differences in β diversity were evaluated using the PERMANOVA Test. OPLS‐DA separation was tested using PERMUTATION Test. Significance levels: n.s., not significant; *p < 0.05; **p < 0.01; ***p < 0.001. CON, control; ABX, preconception antibiotic; PCoA, principal coordinate analysis; KEGG, Kyoto Encyclopedia of Genes and Genomes; OPLS‐DA, Orthogonal Partial Least Squares Discriminant Analysis.

ABX_MA gut microbial community showed reduced α diversity and distinct β diversity (Figure 2A). Some potential pathogens, such as *Klebsiella oxytoca*, *Clostridium innocuum*, *Clostridioides difficile*, *Enterocloster aldenensis*, and *Enterococcus gallinarum* were enriched. In contrast, several beneficial taxa and short‐chain fatty acid (SCFA)‐producing bacteria, including *Firmicutes bacterium M10‐2*, *Lactobacillus intestinalis*, *Lactobacillus johnsonii*, *Bifidobacterium animalis*, and *Bifidobacterium pseudolongum*, were depleted (Figure S3A,B). Functionally, ABX_MA exhibited elevated α diversity and distinct β diversity (Figure 2B), with notable activation of antibiotic resistance pathways (such as biosynthesis of ansamycins, and biosynthesis of vancomycin group antibiotics) and inflammatory pathways (such as lipopolysaccharide biosynthesis, and bacterial invasion of epithelial cells), alongside suppression of basic metabolic functions such as D‐alanine metabolism, ribosome, and secondary bile acid biosynthesis (Figure S3C,D). These observations reflect shifts in the functional composition of the gut microbiome at the maternal stage, as inferred from metagenomic gene profiles. Metabolomic profiling further revealed a clear separation (Figure 2C), with significant accumulation of stress‐related and pro‐inflammatory metabolites (including methyl nicotinate, lithocholic acid, and arachidonic acid) and depletion of protective compounds, including propionate, valerate, indole‐3‐propionic, and hyodeoxycholic acids (Table S1).

The ABX_JU group exhibited persistent alterations in gut microbial communities, with increased α diversity and distinct community composition clustering (Figure 2D). It retained maternal pathogens such as *Klebsiella oxytoca* and *Clostridium innocuum* while acquiring additional taxa like *Akkermansia muciniphila*, *Parabacteroides goldsteinii*, and *Bacteroides stercorirosoris*. In contrast, CON_JU maintained stable colonization by beneficial taxa, including *Firmicutes bacterium M10‐2*, *Bacteroides thetaiotaomicron*, *Parabacteroides merdae*, and *Bacteroides uniformis* (Figure S4A,B). Functionally, ABX_JU exhibited shifts in microbial functional pathways inferred from metagenomic gene profiles, including enrichment of endocrine‐related pathways (such as steroid hormone biosynthesis) and enhanced xenobiotic degradation (such as chlorocyclohexane and chlorobenzene degradation). Conversely, pathways associated with energy metabolism (including oxidative phosphorylation and glycolysis/gluconeogenesis) and development‐related processes (including the Wnt signaling pathway and histidine metabolism) were comparatively suppressed (Figure S4C,D). Metabolomic profiling further revealed a distinct separation (Figure 2F) with elevated levels of pro‐inflammatory metabolites (such as phenol and 3‐Methylindole) and severe depletion of developmental resources, including L‐histidine, citrulline, L‐tryptophan, and phytosphingosine (Table S2).

By adulthood, disruptions in microbial taxonomy, function, and metabolism induced by maternal antibiotic exposure remained evident. In ABX_AD, microbial β diversity differed markedly (Figure 2G), with enrichment of taxa, including *Turicibacter sp. 1E2, Clostridium innocuum*, *Akkermansia muciniphila*, and *Faecalibaculum rodentium*. Conversely, CON_AD retained taxa, such as *Firmicutes bacterium M10‐2* and *Pseudoflavonifractor sp. 524_17* (Figure S5A,B). Functional α diversity decreased, and β diversity showed clear separation (Figure 2H). In ABX_AD, microbial functional pathways inferred from metagenomic gene profiles showed pronounced enrichment in xenobiotic degradation (including chlorocyclohexane and chlorobenzene degradation) and sustained reduction in energy metabolism‐related pathways (such as oxidative phosphorylation) (Figure S5C,D). Metabolomic analysis revealed accumulation of pro‐inflammatory or neurotoxic compounds (such as 2,8‐quinolinediol, deoxycholic acid, and [±]15‐HETE), accompanied by severe depletion of key developmental metabolites (including D‐mannose‐6‐phosphate, L‐thyroxine, Lipoic acid, and *N*‐acetylgalactosamine) (Figure 2I and Table S3).

Notably, across the MA, JU, and AD stages, we identified a range of intergenerationally consistent differential features, defined as those showing significant differences at two or more developmental time points. At the taxonomic level, 25 species demonstrated stable differential patterns across more than two stages (Figure 2J), with taxa such as *Clostridium innocuum* and *Alistipes finegoldii* consistently enriched in the ABX group, whereas *Firmicutes bacterium M10_2* remained dominant in the CON group (Figure S6). At the functional level, 13 pathways showed consistent intergenerational differences (Figure 2K), most notably the sustained activation of chlorocyclohexane and chlorobenzene degradation pathway in the ABX group (Figure S7). Metabolomic analysis revealed 31 metabolites with consistent intergenerational alterations (Figure 2L). Although no metabolite was conserved across all three stages, four metabolites consistently exhibited ABX‐associated enrichment from MA to AD, including oxindole, *N*‐acetyl‐D‐galactosamine, norelgestromin, and 11β‐hydroxyandrostenedione, whereas three metabolites showed persistent enrichment in the CON group, such as oxoadipic acid, arabinose, and desthiobiotin (Figure S8). Moreover, the intergenerationally consistent differential species and metabolites showed strong correlations (Figure S9). Using samples with both sequencing and phenotypic data, we assessed the association between these features and phenotypes at juvenile and adult stages in animal studies. The results showed that many intergenerationally conserved microbial species and metabolites correlated with phenotypes, and several of them showed consistent associations with both JU and AD (Figure 2M). Notably, *Akkermansia muciniphila*, *Alistipes finegoldii*, *Clostridium innocuum*, and *Enterocloster aldenensis* were negatively associated with phenotypes at both stages, whereas *Firmicutes bacterium M10_2* was positively associated with JU and AD indicators. As anticipated, three species that were strongly intergenerationally consistent (showing the same trends across all three stages) also exhibited significant correlations with both JU and AD phenotypes, further supporting their potential role in mediating the long‐term impact of maternal ABX exposure. At the metabolite level, many features were also associated with the phenotypes. A subset of metabolites exhibited concordant correlation trends across JU and AD stages. For example, 1‐naphthylamine and (Z)‐hexadec‐2‐enoic acid were negatively correlated with phenotypes at both stages, whereas 11β‐hydroxyandrostenedione displayed consistent positive associations (Figure 2N).

These findings suggest that preconception antibiotics leave a conserved microbial‒metabolic fingerprint, which propagates alterations in the microbiome from mother to offspring, with specific species and metabolic pathways directly correlated with lifelong gut impairment.

### 2.3 Preconception Antibiotic Exposure Enhanced Maternal‐Offspring Microbial Transmission

**FIGURE 3:**
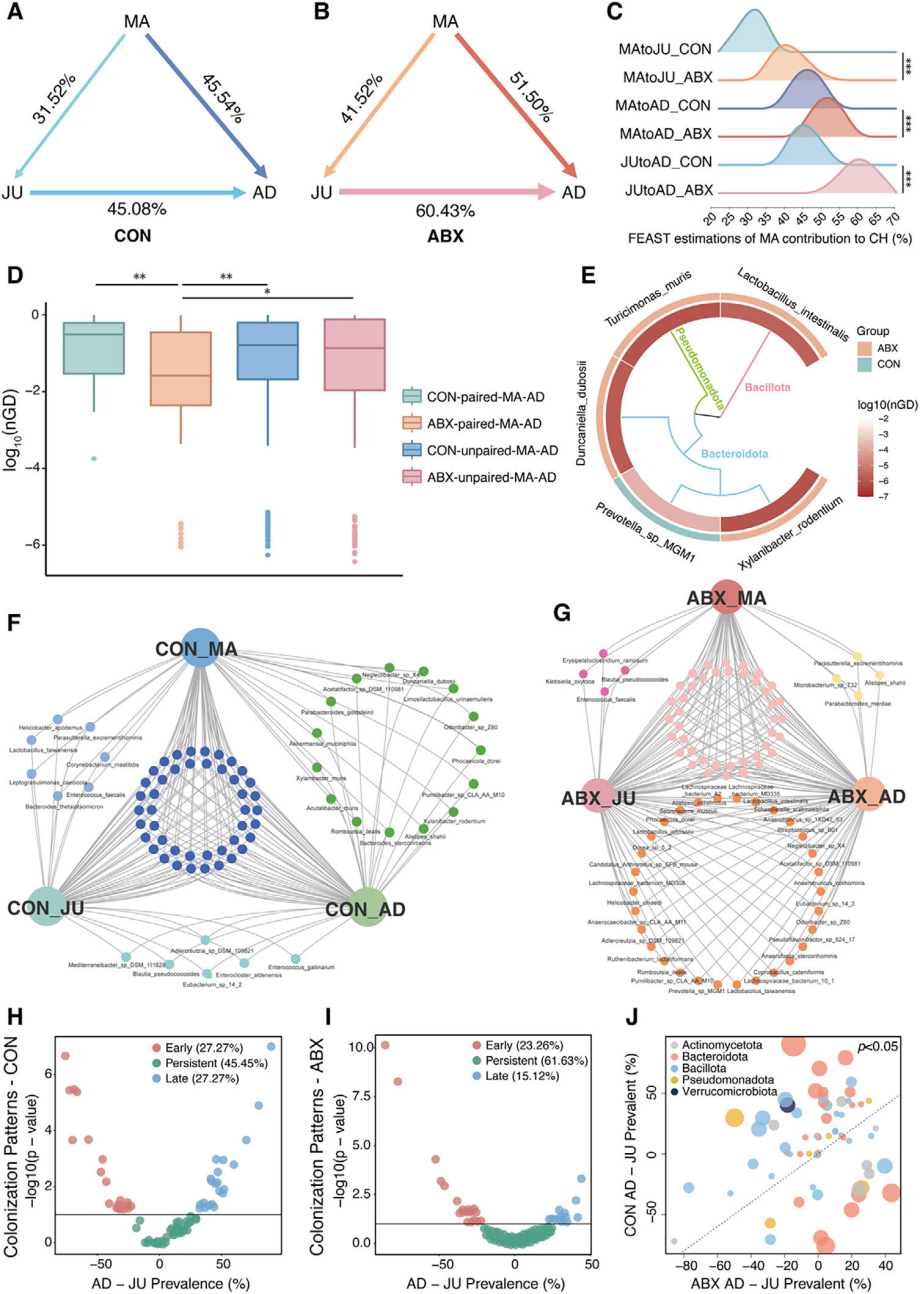
Maternal preconception antibiotics enhanced maternal‐offspring microbial transmission and altered development trajectory. (A,B) Fast expectation‒maximization microbial source tracking (FEAST) analysis at the species level for the CON (A) and ABX (B) groups across developmental stages. Arrows indicate source‐to‐sink directions, with the corresponding percentages denoting the estimated microbial contributions. (C) Ridge plots showing source contribution rate estimates from 10 repeated FEAST runs across developmental stages. (D) Boxplot of log10‐transformed normalized phylogenetic distances (nGD) between maternal (MA) and adult offspring (AD) samples. Comparisons included CON‐paired, ABX‐paired, CON‐unpaired, and ABX‐unpaired MA–AD. (E) Circular heat map of MA and AD strain‐sharing events. Colors represent log10(nGD) values between MA and AD for each shared taxon, with darker shading representing closer strain‐level relatedness. (F‒G) Overlap of microbial species across the three developmental stages in the CON (F) and ABX (G) groups. (H,I) Stage‐specific colonization patterns of species in the CON (H) and ABX (I) groups. Species were categorized as early (significantly higher prevalent in JU), persistent (similar prevalence in JU and AD), or late (significantly higher prevalent in AD). (J) Development trajectory comparing shifts in species prevalence from JU to AD between the CON and ABX groups. Each dot represents a species, and the coordinates reflect the altered developmental trajectory of ABX relative to that of CON. *p*‐values were assessed using the Wilcoxon rank‐sum test with FDR correction. Significance levels: *p < 0.05; **p < 0.01; ***p < 0.001.

These intergenerationally conserved alterations in microbial taxonomy, functional pathways, and metabolite profiles suggest a potential role for microbial transmission in maintaining maternal‒offspring gut microbiota similarity following preconception antibiotic exposure. To verify this hypothesis, we quantified the maternal‐to‐offspring microbial transmission rates using Fast Expectation‐mAximization for microbial Source Tracking (FEAST), which showed that maternal contributions to the offspring gut microbiota were significantly elevated in the ABX group at both JU and AD stages. Specifically, CON_MA contributions to CON_JU and CON_AD were 31.52% and 45.54%, respectively, whereas ABX_MA accounted for 41.52% of JU and 51.50% of AD microbiota. Furthermore, transmission from juvenile to adulthood was also amplified in ABX offspring (ABX_JU→AD: 60.43% vs. CON_JU→AD: 45.08%) (Figure 3A‒C). These results demonstrate the sustained enhancement of maternal‐offspring microbial transmission across developmental stages (MA→JU, MA→AD, and JU→AD) following maternal preconception antibiotic exposure.

To further determine whether the enhanced maternal contribution observed in ABX offspring reflects long‐term strain‐level persistence rather than compositional similarity alone, we conducted strain‐level analysis based on Strainphlan 4. Strain‐level normalized phylogenetic distance (nGD) analysis revealed significantly lower nGD values in the ABX maternal‐adult offspring (MA–AD) pairs compared with both CON pairs and unpaired ABX comparisons, indicating closer strain‐level relatedness specifically in true mother–offspring dyads following preconception antibiotic exposure (Figure 3D). Notably, even in adulthood—long after maternal antibiotic exposure—strain sharing events remained detectable, with more taxa shared in ABX pairs (*Turicimonas muris*, *Xylanibacter rodentium*, *Duncaniella dubosii*, and *Lactobacillus intestinalis*) than in CON pairs (*Prevotella sp MGM1*) (Figure 3E, detailed phylogenies in Figure S10).

Collectively, maternal preconception antibiotic exposure enhances maternal‐offspring microbial transmission and promotes the intergenerational persistence of maternal‐derived taxa, providing a mechanistic basis for the sustained gut perturbation observed in offspring.

### 2.4 Preconception Antibiotic Exposure Altered Offspring Gut Microbial Developmental Trajectory

As described above, preconception antibiotic exposure increases microbial contribution from the juvenile to adult stages. This observation motivated us to investigate whether the juvenile–adult gut microbial developmental trajectory was altered. Analyzing microbial taxonomic dynamics across developmental stages revealed that the ABX group harbored significantly more taxa persisting from juvenile to adult offspring than the CON group (Figure 3F,G), indicating disrupted microbiota maturation.

To better characterize the altered microbiota colonization patterns from juvenile to adult stages, we categorized taxa into three colonization patterns: early colonization (significantly higher prevalence in the JU stage), persistent colonization (similar prevalence in both JU and AD stages), and late colonization (significantly higher prevalence in the AD stage). The ABX group displayed higher persistent colonization than the CON group (61.63% vs. 45.45%), whereas the proportion of late‐colonizing taxa was significantly lower in the ABX group (15.12% vs. CON 27.27%) (Figure 3H,I). This shift contradicts classical ecological succession theory, which posits that stepwise community maturation requires sequential microbial recruitment. However, the ABX microbiota exhibited premature stabilization.

Furthermore, community‐wide trajectory analysis revealed a significant divergence between the CON and ABX groups (Figure 3J). Specifically, many taxa in the ABX group exhibited an “accelerated” pattern, clustering above the *y* = *x* line, indicating that the juvenile microbiota prematurely acquired adult‐like features. A direct comparison of colonization patterns further supported this result: 64.15% of persistent colonizers in ABX were distinct from CON, and these “accelerated” taxa showed maximal overlap (28.30%) with CON late colonizers (Figure S11).

Overall, maternal preconception antibiotic exposure causes premature stabilization of offspring gut microbiota, truncating natural successional processes. This acceleration disrupts host‒microbiota co‐maturation, potentially explaining the persistent alterations in the microbiome and elevated disease susceptibility.

### 2.5 Preconception Antibiotic Exposure Imposed Heightened Selective Pressures During Microbial Community Assembly

**FIGURE 4:**
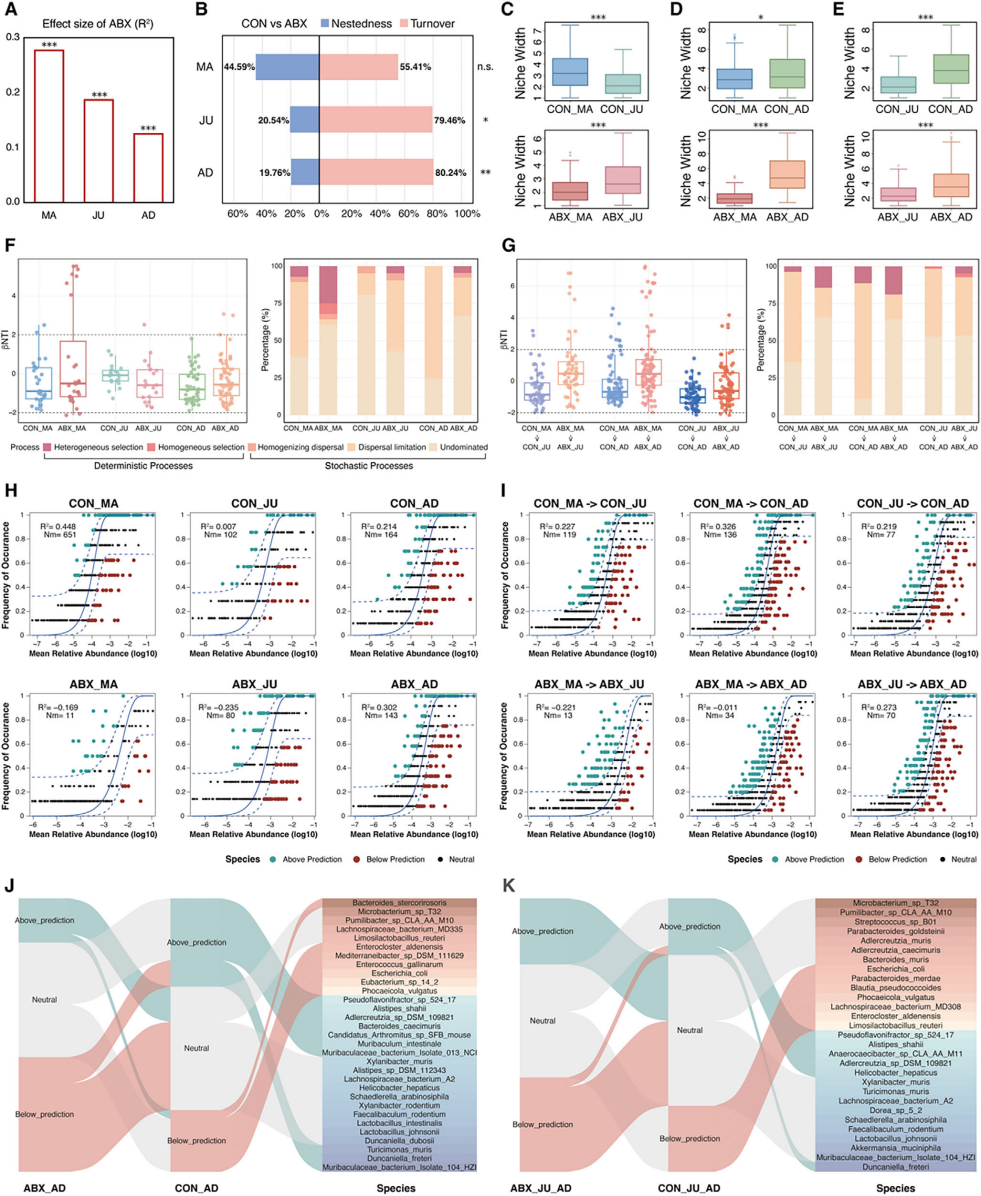
Maternal preconception antibiotic exposure alters microbial community structure, successional dynamics, and assembly mechanisms. (A) Effect sizes (*R* ^2^) of maternal preconception antibiotic exposure on gut microbial composition at each developmental stage (MA, JU, AD), determined by PERMANOVA using Aitchison distance matrices. (B) Partitioning of β diversity into nestedness and turnover at each developmental stage, assessed by Permutation tests (999 permutations). (C–E) Niche breadth (based on Levins index) of shared species between stage pairs: MA and JU (C), MA and AD (D), and JU and AD (E). *p*‐values were calculated using the Wilcoxon rank‐sum test. (F,G), Ecological processes in microbial community assembly within individual stages (F) and across developmental transitions (G). In (F), ecological processes were evaluated within each group using samples from individual developmental stages. In (G), samples from two stages were combined to assess the underlying ecological processes during developmental transitions. Deterministic processes were defined as |βNTI| > 2, and stochastic processes as |βNTI| ≤ 2. (H,I), Neutral community model (NCM) fitted to microbial communities within individual stage (H) and across developmental transitions (I). Solid blue lines represent the best fit to the NCM; dashed lines indicate 95% confidence intervals. Species occurring more (green, above prediction) or less (red, below prediction) frequently than expected under neutrality are indicated. *R* ^2^ denotes model fit; Nm reflects metacommunity size × immigration. (J) Differential dispersal abilities of shared species between ABX‐AD and CON‐AD groups, based on NCM fitting. Species with higher dispersal in ABX‐AD (red) or lower dispersal (green) were identified relative to CON‐AD. (K) Similar comparison of species‐specific dispersal abilities between JU and AD stages in CON and ABX groups. Significance levels: n.s. not significant; *p < 0.05; **p < 0.01; ***p < 0.001.

Following the observed increase in maternal‐offspring microbial transmission and acceleration of microbial developmental trajectories, we further investigated how these changes are reflected at microbial community level. To systematically inspect how maternal preconception antibiotic exposure reshapes microbial ecology, we integrated community structure analysis, assembly mechanism quantification, and taxon‐specific dispersal profiling. Overall, antibiotic exposure induced a significant separation of CON and ABX communities across all stages, with the strongest effects in dams (MA) and progressively attenuated but persistent effects in the offspring (Figure 4A). Further partitioning of intergroup dissimilarity (Figure 4B) revealed that microbial community divergence at the MA stage was driven by both species turnover (55.41%) and nestedness (44.59%), whereas the JU and AD stages were predominantly governed by species turnover (JU: 79.46% vs. 20.54%; AD: 80.24% vs. 19.76%), indicating that antibiotic‐induced changes in the offspring microbiome are not solely due to the loss of specific taxa but also involve active species replacement. Having established the persistent divergence between the CON and ABX communities across developmental stages, we sought to understand how antibiotic exposure alters the niche width dynamics within each group over time. Niche dynamics of the shared taxa were identified across paired time points (MA→JU, JU→AD, and MA→‒AD). In the CON group, MA taxa exhibited a broader niche breadth than JU, whereas the reverse pattern was observed in the ABX group, where JU taxa showed broader niches than MA taxa, indicating an antibiotic‐induced maternal niche contraction (Figure 4C). For MA→AD and JU→AD transitions, both CON and ABX groups showed niche expansion from MA/JU to AD, with ABX exhibiting more pronounced increases (Figure 4D,E).

These consistent shifts in community structure and niche dynamics raise the question of whether preconception antibiotic exposure alters the ecological processes governing microbial community assembly. To investigate this, we applied a null model analysis to assess the relative contributions of deterministic and stochastic processes. Null model analysis confirmed that deterministic processes were consistently stronger in the ABX group across both single stages (MA, JU, AD, Figure 4F) and transitional stages (MA→JU, MA→AD, JU→AD, Figure 4G). Deterministic processes refer to species sorting driven by environmental selection pressure (including antibiotic pressure), whereas stochastic processes consider these changes as random ecological drifts. Building on these group‐level observations, the neutral community model was employed to evaluate species‐level deviations from neutral expectations, providing a more detailed perspective on how selective pressures shape microbial assembly. ABX communities, at both single stages (ABX_MA and ABX_JU) and in transitional comparisons (ABX_MA → ABX_JU and ABX_MA → ABX_AD), deviated from neutral predictions (*R* ^2^ < 0; Figure 4H,I), whereas CON communities generally conformed to neutral expectations, indicating that selective pressures override random drift in species dynamics of ABX. Notably, in the AD stage communities, ABX high‐dispersal taxa (vs. CON) were pro‐inflammatory or pathogenic, such as *Bacteroides stercorirosoris*, *Enterocloster aldenensis*, and *Enterococcus gallinarum*, whereas ABX low‐dispersal taxa included SCFA‐producing or probiotic species, such as *Lactobacillus intestinalis, Lactobacillus johnsonii*, *Alistipes shahii*, and *Xylanibacter rodentium* (Figure 4J,K). Co‐occurrence network analysis further revealed that low‐dispersal taxa engaged more in competitive (negative) interactions, whereas high‐dispersal taxa formed more cooperative (positive) networks with other taxa in ABX_AD (Figure S13).

These results highlight that maternal antibiotic exposure amplifies deterministic selection in offspring microbial assembly. This assembly shift established a deterministic framework for persistent alterations in microbiome and disease susceptibility in offspring.

### 2.6 Functional Validation: Microbiota‐Mediated Colitis Susceptibility via Fecal Microbiota Transplantation (FMT)

**FIGURE 5:**
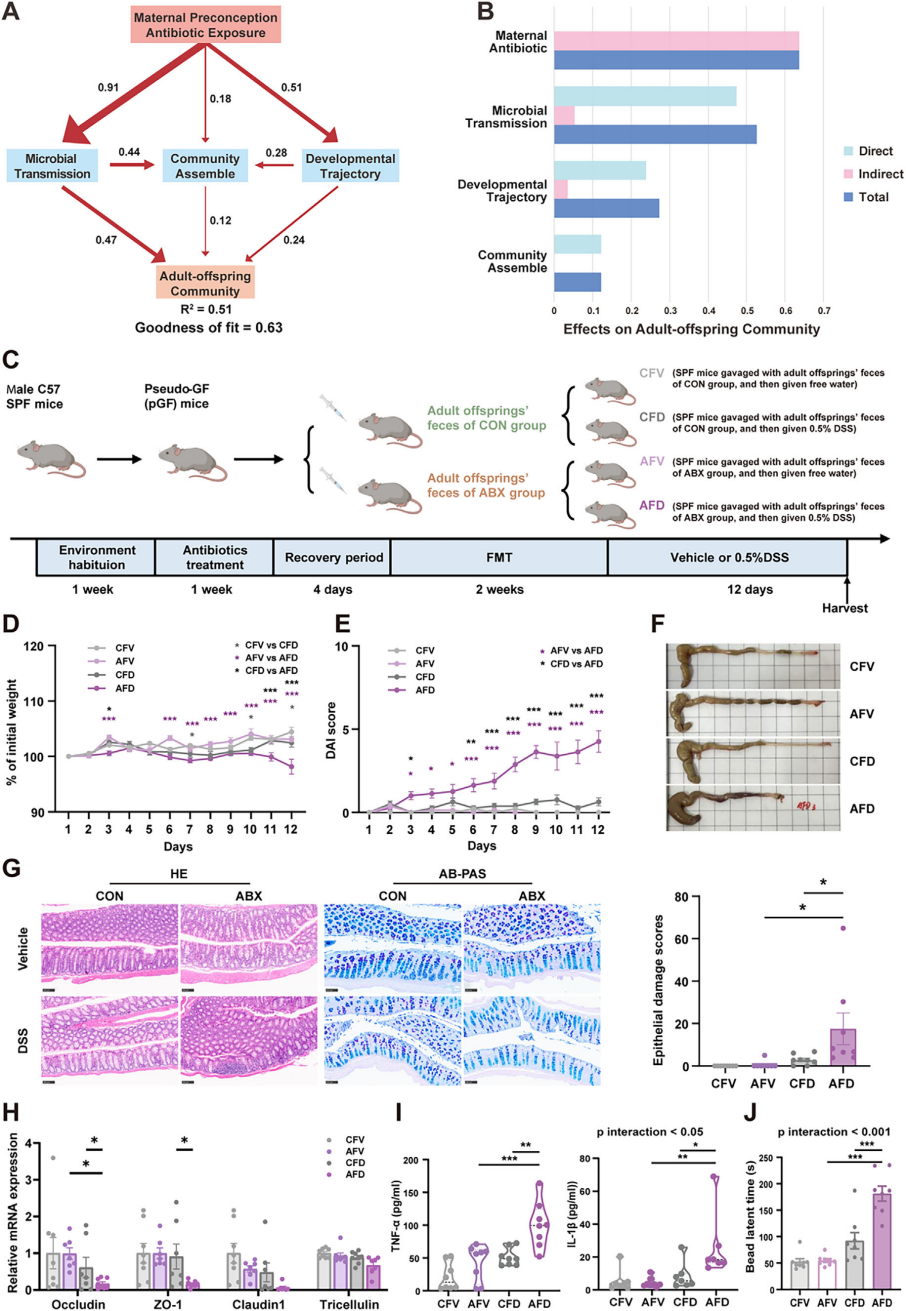
Through a cascade of disrupted microbial succession, maternal preconception antibiotic exposure induces persistent shifts in offspring microbial community that functionally drive increased colitis susceptibility, as supported by fecal microbiota transplantation. (A) Partial least squares path modeling (PLS‐PM) depicting the structural relationships among maternal preconception antibiotic exposure, enhanced maternal‐offspring microbial transmission, altered developmental trajectory, increased selective constraints in community assembly, and adult offspring microbiota. Path coefficients are represented by arrow widths and are annotated with detailed data. (B) Standardized direct, indirect, and total effects of maternal preconception antibiotic exposure on adult offspring microbial community, as estimated by PLS‐PM. (C) Schematic of the experimental design: Male mice were treated with antibiotics to establish a pseudo‐sterile state, received FMT from adult offspring of the CON and ABX groups, and were administered either sterile water or 0.5% DSS solution for 12 consecutive days. (D) Normalized percentage weight loss of mice during administration of sterile water or 0.5% DSS solution (*n* = 8). (E) Disease activity index (DAI) scoring of mice during administration of sterile water or 0.5% DSS solution (*n* = 8). (F) Representative colon images of mice after administration of sterile water or 0.5% DSS solution. (G) Representative images of hematoxylin & eosin and Alcian Blue‐Periodic Acid Schiff staining of the colon (scale bar = 100 µm). Histopathological scores after administration of sterile water or 0.5% DSS solution (*n* = 8). (H) Relative mRNA expression of colonic barrier‐related genes (Occludin, ZO‐1, Claudin1, and Tricellulin) (*n* = 7‒8). (I) Concentrations of inflammatory factors (TNF‐α and IL‐1β) (*n* = 8). (J) Colonic transport time (*n* = 8). Data are presented as mean ± SEM. Two‐way repeated measures ANOVA followed by Sidak's multiple comparison test was used (D, E). Two‐way ANOVA followed by Tukey's (G, I, J) or Sidak's (H) multiple comparison test was used for analyses involving two variables. *p < 0.05, **p < 0.01, ***p < 0.001.

To test whether the altered microbiota of adult offspring—resulting from maternal preconception antibiotic exposure—is itself sufficient to confer increased susceptibility to colitis. Using partial least squares path modeling (PLS‐PM), we quantified the cascading effects of maternal preconception antibiotic exposure on the gut microbial community of the offspring. The results revealed that antibiotic exposure was positively associated with the enhanced maternal‐offspring microbial transmission, altered developmental trajectory of the gut microbiota, and imposed heightened selective pressure on community assembly. These factors collectively contributed to persistent shifts in the microbiome of adult offspring (Goodness of Fit = 0.63, Figure 5A). Among these factors (Figure 5B), enhanced maternal‐offspring microbial transmission had the greatest direct impact on the microbial community of adult offspring (standardized effect size = 47.35%), followed by altered developmental trajectory (23.86%) and increased deterministic assembly (12.15%). This model confirmed that the disrupted microbiota in offspring was fundamentally rooted in maternal preconception exposure to antibiotics.

To investigate whether the altered gut microbiome directly increases the risk of colitis, we transplanted fecal microbiota from CON/ABX adult offspring into pseudo‐sterile mice (FMT). These mice were then subjected to a 0.5% DSS challenge (CFD and AFD groups) or vehicle control (CFV and AFV groups) (Figure 5C). AFD recipients (ABX‐FMT + DSS) exhibited significantly more severe colitis than controls (CFD and AFV groups), as evidenced by greater weight loss, higher DAI scores, and colon shortening (Figure 5D‒F and Figure S14A). Further, the AFD group presented a reduction in goblet cell numbers and compromised epithelial integrity (Figure 5G and Figure S14B), along with decreased expression of the barrier genes Occludin and ZO‐1 (Figure 5H), compared with the CFD and AFV groups. Moreover, TNF‐α and IL‐1β levels were significantly elevated in the AFD group (Figure 5I and Figure S14C). Consistently, the AFD group showed impaired colon motility, although this effect was not observed in the small intestine (Figure 5J and Figure S14D).

This integrated approach not only identifies a statistical association (via PLS‐PM) but also provides biological support (via FMT), collectively suggesting a potential microbiota‐mediated mechanism that underlies the intergenerational transmission of disease risk.

### 2.7 Preliminary Clinical Exploration: Disrupted Microbial Succession in PIBD Possibly Associated With Maternal Antibiotic Exposure

**FIGURE 6:**
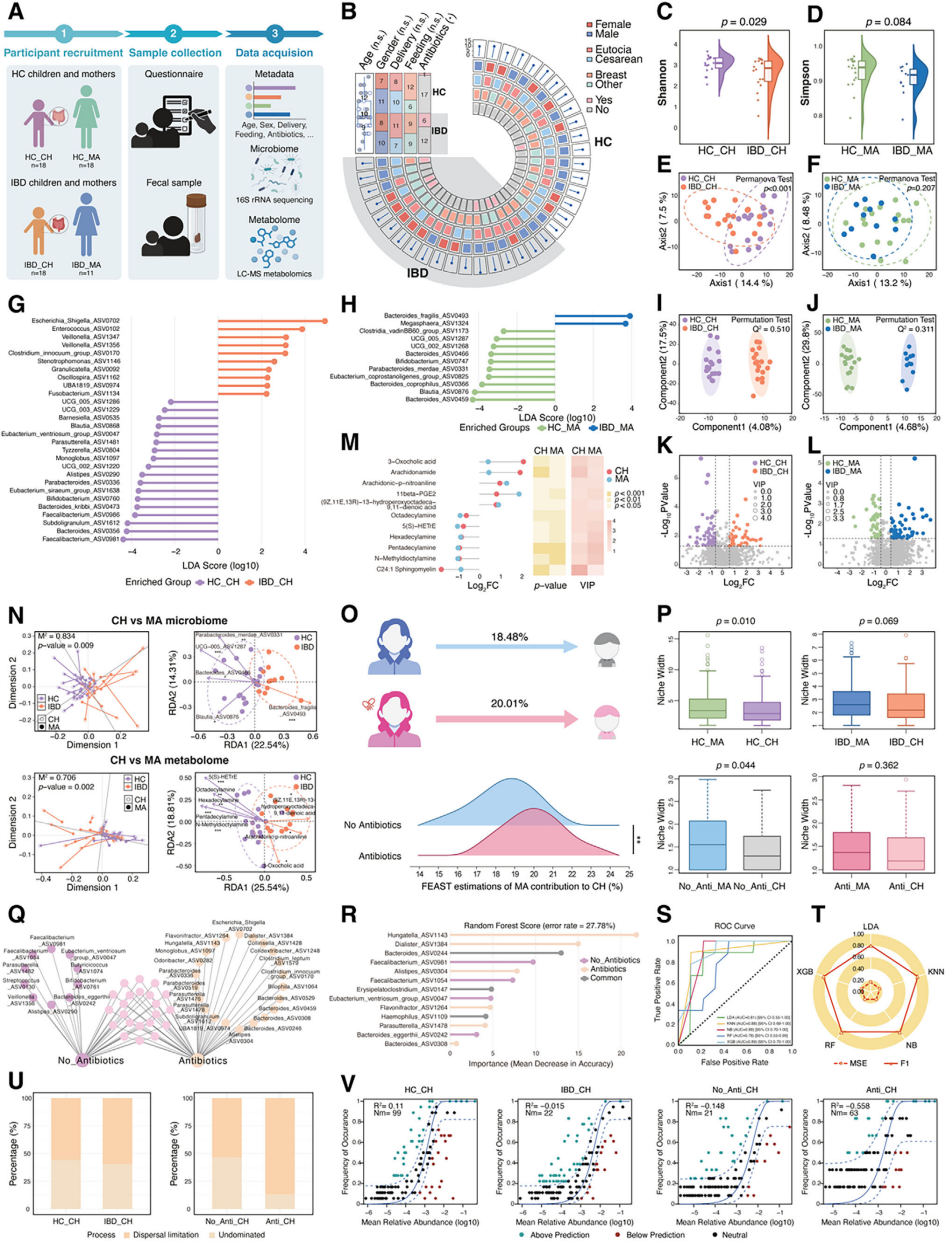
Case‒control study suggests that maternal antibiotic exposure is associated with promotes mother‒child microbial transmission and selective pressures in shaping offspring microbiota. (A) Schematic of study design. Metadata and stool samples were collected from 18 children with inflammatory bowel disease and their mothers (*n* = 11), and 18 healthy control children and their mothers (*n* = 18). (B) Summary of demographic characteristics. (C‒D) α diversity of gut microbiota at the ASV level in children (Shannon index, C) and mothers (Simpson index, D). Variations were assessed using the Wilcoxon rank‐sum test. (E,F) Principal coordinate analysis (PCoA) based on Aitchison distance at the ASV level in children (E) and mothers (F). Differences were assessed using the PERMANOVA test. (G,H) Linear discriminant analysis Effect Size (LEfSe) of differential microbial taxa (ASV) in children (G) and mothers (H); taxa with LDA score >2 and Wilcoxon rank‐sum test *p* < 0.05 are shown. (I,J) Orthogonal Partial Least Squares Discriminant Analysis (OPLS‐DA) plots of gut metabolome in children (I) and mothers (J). (K,L) Volcano plots of differential metabolites in children (K) and mothers (L). (M) Shared altered metabolites with consistent trends in children with IBD and their mothers. (N) Procrustes and Redundancy analyses reveal a significant correlation between maternal and child microbial and metabolite profiles. (O) Fast Expectation‐mAximization for microbial Source Tracking (FEAST) analysis, estimating the contribution of maternal microbiota to child gut communities under different antibiotic exposure contexts. Ridge plots show the distribution of maternal contributions across 20 iterations, with *p*‐values calculated using the Wilcoxon rank‐sum test and FDR correction. (P) Comparison of maternal and child microbial niche breadth under different stratifications (including IBD vs. HC; Antibiotic‐exposed vs. Unexposed), using shared ASVs in paired groups. *p*‐values were also assessed using the Wilcoxon rank‐sum test. (Q) Comparison of shared ASVs between mother‒child pairs in the presence or absence of maternal antibiotic exposure. (R) Random Forest identifying key shared ASVs distinguishing antibiotic vs. non‐antibiotic mother‒child pairs; antibiotic‐specific species ranked with higher feature importance. (S) Receiver operating characteristic curve of five machine learning models (Linear Discriminant Analysis, K‐Nearest Neighbors, Naïve Bayes, Random Forest, and eXtreme Gradient Boosting) in distinguishing antibiotic exposure status of mother–child pairs based on key shared ASVs. (T) Model performance evaluation using mean squared error (MSE) and F1 score. (U) Ecological processes shaping microbial community assembly in child cohorts: comparison between healthy children (HC_CH) and children with IBD (IBD_CH), and between maternal‐antibiotic‐exposed (Anti_CH) and unexposed (No_Anti_CH) children. (V) Neutral community model (NCM) fitting for gut microbial communities in different groups. Solid lines represent model fit; dashed lines indicate 95% confidence intervals; *R* ^2^ indicates goodness‐of‐fit to NCM. Significance levels: n.s. not significant, ·*p* < 0.1, *p < 0.05, **p < 0.01, ***p < 0.001.

To assess whether the potential mechanisms identified in the animal models are applicable to humans, we established a clinical cohort. Given the formidable challenge of prospectively collecting samples from mother–child pairs where the mother had preconception antibiotic exposure and the child later developed IBD, we opted for a case‒control study design (Figure 6A). This study included 18 pediatric patients with IBD (IBD_CH) and their mothers (IBD_MA), as well as 18 demographically matched healthy child–mother pairs (HC_CH, HC_MA). Despite comparable demographics (child age, sex, delivery mode, and feeding practices) between the groups, perinatal antibiotic exposure was notably higher among mothers with IBD (Figure 6B and Table S4).

As expected, children with IBD showed pronounced alterations in the gut microbial community compared with healthy controls. α diversity was significantly lower (Figure 6C and Figure S15A), and β diversity revealed distinct microbial communities between IBD_CH and HC_CH (Figure 6E). Linear discriminant analysis Effect Size (LEfSe) highlighted the enrichment of potentially harmful or opportunistic bacteria such as *Escherichia* *Shigella, Enterococcus*, and *Clostridium innocuum*, and the depletion of beneficial taxa such as *Faecalibacterium, Bacteroides*, and *Bifidobacterium* (Figure 6G). Importantly, a subset of these differentially abundant taxa remained significant in the multivariate association analyses after adjusting for key covariates (including age, gender, delivery, and feeding), as assessed by MaAsLin2 (Figure S15D), supporting the robustness of these disease‐associated microbial signatures. Metabolomic profiling also revealed distinct differences (Figure 6I), with differential metabolites primarily involved in amino acid, fatty acid, and benzene derivative metabolism. KEGG pathways such as ABC transporters, riboflavin, and thiamine metabolism were suppressed in IBD_CH (Figure 6K and Figure S16A,C, Table S5). Notably, even the mothers of children with IBD, who appeared healthy, exhibited distinct microbial and metabolic profiles. α diversity was reduced in IBD_MA compared with HC_MA (Figure 6D and Figure S15B), although β diversity did not reveal a significant overall separation (Figure 6F). LEfSe showed a higher abundance of *Bacteroides fragilis* and reduced levels of health‐associated taxa, such as *Bacteroides* and *Bifidobacterium*, in the IBD_MA group (Figure 6H). These associations were further supported by multivariate analysis after adjusting for available maternal covariates (Figure S15E). Despite the lack of an overall separation, metabolomic differences were observed (Figure 6J,L and Table S6), primarily involving benzene derivatives, organic acids, and amino acids with enhanced pathways, including cholesterol and arachidonic acid metabolism (Figure S16B,D). Notably, both children and mothers shared inflammatory metabolic signatures, characterized by elevated pro‐inflammatory markers (such as arachidonamide, arachidonic‐p‐nitroaniline, and 11β‐PGE2) and reduced anti‐inflammatory metabolites (such as 5(S)‐HETrE) (Figure 6M).

The Mantel test suggested a strong coupling between differential microbes and metabolites in both children (Figure S17A) and mothers (Figure S17B). Procrustes analysis supported a strong correlation between IBD‐associated microbial signatures in children and their mothers. Redundancy analysis identified key maternal taxa driving microbiome shifts in children, including *Bacteroides fragilis*, *UCG‐005*, *Parabacteroides merdae*, *Blautia*, and *Bacteroides*. Similarly, maternal metabolites such as (9Z, 11E, 13R)‐13‐hydroperoxyoctadeca‐9,11‐dienoic acid, arachidonic‐p‐nitroaniline, and 5(S)‐HETrE were major predictors of metabolic shifts in children with IBD (Figure 6N).

Given the higher rate of perinatal antibiotic use among mothers of children with IBD and our specific interest in the role of maternal antibiotics in offspring colitis susceptibility, we established an IBD sub‐cohort (antibiotic‐exposed, *n* = 6; unexposed, *n* = 6) matched for disease severity, delivery mode, and feeding practices to further explore these potential mechanisms in humans. Mothers exposed to antibiotics transmitted significantly more microbes to their children (Figure 6O). Although healthy children and their mothers exhibited significant differences in niche width, this was not observed in the IBD dyads, primarily because of the antibiotic‐exposed group, where maternal and child niche widths were similar (Figure 6P). Intriguingly, maternal antibiotic exposure not only enhanced microbial transmission but also shaped distinct mother–child‐shared microbiota profiles. Antibiotic‐exposed dyads shared more antibiotic‐resistant taxa or potentially pathogenic bacteria, such as *Escherichia Shigella*, *Clostridium innocuum*, and *Flavonifractor* (Figure 6Q). Random Forest feature importance confirmed that exposure‐specific shared microbes (as opposed to overlapping taxa) were the primary classifiers of maternal antibiotic history (Figure 6R). Using these biomarkers, machine learning models (Linear Discriminant Analysis, K‐Nearest Neighbors, Naïve Bayes, Random Forest, and eXtreme Gradient Boosting) robustly diagnosed exposure (area under the curve: 0.78‒0.89; mean squared error: 0.17; F1: 0.80‒0.86) (Figure 6S,T). The community assembly process differed slightly between the children with IBD and healthy children. However, antibiotic exposure in children with IBD contributed to a marked shift toward dispersal limitation, in contrast to unexposed children (Figure 6U). The neutral community model analysis further showed that the deviation from neutrality in children with IBD was largely driven by the antibiotic‐exposed group, in which the species dynamics strongly deviated from stochastic expectations (Figure 6V).

Collectively, our clinical cohorts demonstrated that IBD child‒mother dyads exhibit intergenerationally coupled perturbations in the gut microbiome, with maternal antibiotic exposure being associated with enhances microbial transmission routes and promotes selective pressure in community assembly.

## 3 Discussion

By integrating animal models with preliminary clinical exploration, our study reveals that maternal antibiotic exposure during the preconception period exerts a significant and long‐lasting impact on offspring health. Through a cascade of mechanisms, including enhanced maternal‐offspring microbial transmission microbes, altered microbial developmental trajectories, and increased selective pressure during community assembly, maternal microbiome perturbations disrupt the offspring gut microbiome, impair gut development, and increase susceptibility to IBD.

### 3.1 Observation: Maternal Preconception Antibiotic Exposure Induces Transgenerational Gut Mucosal Immaturity, Colitis Susceptibility, and Microbiome Perturbations

Animal experiments have demonstrated that maternal preconception antibiotic exposure impairs intestinal development and increases the susceptibility to colitis in offspring. Although several clinical and animal studies have identified the impact of antibiotic exposure during pregnancy on IBD or colitis [21, 22], our study uniquely extended this timeframe to the preconception period. This discovery redefined the critical window for development and underscored the importance of preconception health in mitigating long‐term disease risk [23]. Notably, offspring from antibiotic‐exposed dams did not develop spontaneous colitis but exhibited impaired colonic mucosa and barrier function, making them highly susceptible to the disease under minimal stimuli (0.5% DSS). These findings are likely relevant to human IBD, where at‐risk individuals may be more sensitive to factors that trigger an inflammatory response, leading to an overt clinical disease [24, 25].

Moreover, our study indicated that maternal preconception antibiotic exposure causes persistent alterations in the gut microbiome. In dams, antibiotic exposure led to a high prevalence and significant enrichment of multiple potentially pathogenic and drug‐resistant bacteria, such as *Klebsiella oxytoca*, *Clostridium innocuum*, and *Clostridioides difficile*, reflecting the potent disruption effect of antibiotics on their microbiota [26]. This disruption persisted in juvenile and adult offspring. The ABX group was still dominated by pathogenic bacteria, although the specific differential bacteria varied, and the adult microbial disorder partially normalized. More importantly, we identified a range of intergenerationally consistent differential microbial features, such as *Clostridium innocuum* and *Alistipes finegoldii*, consistently enriched in the ABX group, and *Firmicutes bacterium M10_2* persistently present in the CON group. These features also exhibited significant and consistent correlations with the juvenile and adult phenotypes. *Clostridium innocuum* causes extraintestinal infection and antibiotic‐associated diarrhea [27]. Recent studies have found that *Clostridium innocuum* is associated with poorer clinical remission in patients with ulcerative colitis (UC) [28] and can promote the formation of creeping fat and intestinal strictures in patients with Crohn's disease [29]. Similarly, a recent clinical study reported that *Alistipes finegoldii* was positively associated with disease activity in adult patients with UC [30]. Additional experimental evidence supports its pro‐inflammatory potential, as short‐term colonization (oral administration for 1 week) induced intestinal inflammation in both wild‐type (WT) and IL10^−^/^−^ C57BL/6J mice [31]. Importantly, both *Clostridium_innocuum* and *Alistipes finegoldii* exhibit resistance to multiple antibiotics, including vancomycin, kanamycin, and colistin [32, 33], which may underlie their sustained presence in the ABX lineage and facilitate their expansion during microbial succession.

Consistent with alterations in the gut microbiome, the maternal preconception of antibiotic exposure also leads to persistent functional and metabolic perturbations. At the functional level, the most notable intergenerational difference was the sustained activation of the chlorocyclohexane and chlorobenzene degradation pathways in the ABX group. Chlorocyclohexane and chlorobenzene are hazardous organic compounds that adversely affect human health [34], and the structurally related dinitrochlorobenzene has been shown to induce colitis in experimental animal models [35]. Nie et al. found that the presence of the chlorocyclohexane and chlorobenzene degradation pathway in the gut microbiota may be correlated with sleep efficiency [36]. The continuous activation of this pathway in the ABX group over three generations demonstrated that maternal antibiotic exposure led to the persistent presence of harmful substances in the offspring. Compared with alterations in the gut microbiota, changes in metabolites were relatively inconsistent, as evidenced by the lack of significant separation between ABX and CON in adulthood, no metabolites showing consistent alteration across all three stages, and inconsistent metabolite–phenotype correlations in juvenile and adult offspring. The weaker generational consistency observed in fecal metabolomic profiles likely arises from a combination of biological and technical factors. (1) Unlike microbial taxonomic composition, fecal metabolites represent an integrated output of multiple interacting sources, including microbial metabolism, host intestinal secretion and absorption, dietary inputs, and luminal transformation. As a result, metabolite profiles are inherently more context‐dependent and subject to dynamic regulation, which may attenuate uniform associations across developmental stages and generations. (2) Although untargeted metabolomics provides a broad survey of the metabolic landscape, it may be less sensitive than high‐resolution microbial sequencing in detecting subtle, lineage‑consistent shifts, especially across varying developmental stages. These findings further demonstrate that compared to metabolites, the successional dynamics of the gut microbiota plays a more significant role in individual development.

### 3.2 Potential Mechanism: Disrupted Microbial Succession Sustains Microbiome Perturbations and Impacts Offspring Colitis Susceptibility

It is well established that antibiotic exposure induces gut microbiome perturbation [37, 38, 39]. Consistent with this, our findings demonstrate that maternal antibiotic exposure before conception, even with a recovery window, led to sustained perturbation characterized by a compositional imbalance, such as enrichment of opportunistic pathogens. Furthermore, our results revealed that preconception antibiotic exposure significantly enhanced maternal‐offspring microbial transmission, with ABX mothers contributing more to both juvenile and adult offspring communities than controls. Notably, strain‐level analyses revealed that even long after maternal preconception antibiotic exposure, ABX offspring retained more maternally shared strains than controls, highlighting persistent intergenerational transmission. As previously proposed, early‐life gut microbiota serves as the foundational inoculum that shapes the niche for subsequent species and profoundly influences lifelong microbial communities and host health [40, 41]. Thus, this dual impact, maternal gut microbiome shift combined with the enhanced transmission of maternally derived microbes, may establish a perturbed initial community, thereby underlying the persistent alteration observed in ABX offspring.

Furthermore, preconception antibiotic exposure appeared to promote sustained microbial presence from juvenile to adult stages. In the ABX offspring group, more taxa persisted across stages without typical turnover, whereas fewer new microbes were recruited during the adult phase compared with controls. This altered pattern suggests premature stabilization of the gut ecosystem, potentially caused by early life over‐colonization by maternal‐origin microbes, or a constrained ecological niche that impairs natural progression toward adult‐associated taxa. From an ecological perspective, this flattening of microbial succession runs counter to the expected age‐dependent diversification seen in healthy development, where increasing complexity and compositional shifts are hallmarks of microbial and host co‐maturation [42, 43]. Such accelerated, non‐synchronous colonization may impair the temporal coordination between microbial development and host physiological programming, potentially affecting immune tolerance and barrier function during the critical windows of postnatal development [44, 45].

Beyond disrupting microbial transmission and maturation, the preconception antibiotic exposure has imposed lasting and widespread shifts in community structure and assembly dynamics across generations. Further dissection of β diversity components revealed that offspring microbial differences were primarily driven by species turnover rather than nestedness, implicating active replacement of taxa rather than mere loss [46]. These findings suggest that maternal preconception antibiotic exposure does not simply eliminate microbes but actively reshapes successional pathways, ultimately establishing a noncanonical microbial community in offspring. Consistently, the null model analysis supported a stronger role for deterministic processes in the ABX group (versus the CON group) across both intra‐ and inter‐stage transitions, indicating that microbial assembly in ABX is more tightly governed by selective pressure rather than neutral drift [46, 47, 48]. Such heightened selective pressure may arise from preconception‐antibiotic‐induced alterations along the maternal‐offspring axis, including biased microbial transmission, reduced niche availability, impaired host physiology, and disrupted immune regulation. Importantly, although stochastic processes appeared to recover in adulthood, long‐lasting consequences persisted: taxa with elevated dispersal ability in ABX‐AD were enriched in pro‐inflammatory lineages, whereas low‐dispersal taxa included beneficial SCFA producers and probiotics, such as *Lactobacillus intestinalis* and *Lactobacillus johnsonii* [49, 50, 51]. Network analysis further revealed that low‐dispersal beneficial taxa were embedded in dense negative interactions, potentially reflecting competitive exclusion or suppression by dominant pathobionts. Conversely, highly dispersed pro‐inflammatory taxa showed expanded positive interactions, suggesting ecological dominance and cooperative expansion [52]. Although our study identified potential microbial mechanisms and taxa of interest, the analyses provided descriptive and associative insights. Their causal roles require further experimental validation in future study.

Given that the disrupted microbial succession led to disorders of the gut microbiota, we conducted an FMT experiment to further support the impact of preconception antibiotic exposure‐induced microbiome changes in the offspring on colitis. As for pseudo‑sterile mice display a relatively normal—albeit attenuated—inflammatory response and are less prone to hemorrhage and epithelial injury than germ‐free mice, we adopted pseudo‑sterile model in FMT experiment. The results clearly demonstrated that the disorder of gut microbiota in the offspring caused by antibiotic exposure itself, rather than the off‐target effect of antibiotics or other factors, was the core pathogenic factor driving the increased susceptibility to colitis. Even in adulthood, antibiotic‐induced microbial disorders retain the capacity to directly remodel the intestinal microenvironment and compromise homeostasis, which is sufficient to significantly increase the susceptibility of recipient animals to DSS damage.

### 3.3 Translational: Human Data Suggest Similar Microbial Succession Patterns

Due to the difficulty in collecting samples from mothers who took antibiotics specifically during the preconception period and whose children later developed IBD within such a short time, we conducted a case–control study involving children with IBD and their mothers, as well as healthy children and their mothers. Clinical data echoed our experimental findings; children with IBD exhibited typical gut microbiome perturbations, consistent with existing findings [53, 54], while their mothers, although clinically healthy, also showed parallel disruptions in gut microbiota and metabolites. Notably, we observed a higher proportion of maternal antibiotic exposure during the perinatal period in the IBD group, which is consistent with the findings of previous studies [21, 22, 55], further highlighting the impact of antibiotic use. Crucially, antibiotic‐exposed mothers transmitted significantly more microbes to their children, including antibiotic‐resistant and potentially pathogenic taxa, such as *Escherichia Shigella* and *Bacteroides fragilis* [56, 57]. Shared microbial features between mother and child pairs robustly distinguished maternal antibiotic history, reinforcing the long‐lasting imprint of maternal antibiotic exposure. Moreover, community assembly patterns in antibiotic‐exposed children also shift toward more selective pressure, consistent with our animal findings, suggesting that microbial birth, death, and replication are increasingly governed by selective pressures rather than random drift [58]. It should be noted that the use of hospital‐based healthy controls may introduce selection bias, as these individuals could differ systematically from the general population in health behaviors and baseline exposures. Future studies should employ community‐based controls to better validate the findings.

Collectively, our study is the first to reveal that maternal preconception antibiotic exposure sets off a chain‐reaction microbial succession. This alteration is associated with enhanced maternal‐offspring microbial transmission, altered microbial developmental trajectories, and intensified ecological selection pressure. In combination, these factors result in long‐lasting impairment in the establishment and maturation of the offspring's gut ecosystem. Such impairments not only hamper intestinal development but also substantially elevate the lifelong susceptibility of offspring to inflammatory disorders, such as colitis. Our research serves a dual purpose: it provides a scientific basis for the early identification of at‐risk individuals and offers a promising strategy for microbiota‐based interventions. These interventions, targeting the maternal preconception period, aim to safeguard offspring health. Despite these strengths, this study has some limitations. Our study focuses on shifts in microbial succession rather than delineating the precise routes of vertical transmission. Future studies could adopt more rigorous designs—such as cross‐fostering, early‐life fecal microbiota transplantation, embryo transfer, earlier and more frequent longitudinal sampling—to clarify fine‐scale transmission pathways. Additionally, the clinical study was retrospective and involved a limited sample size, rendering it exploratory and preliminary in nature. As such causal interpretations are limited, underscoring the need for prospective birth cohort studies. Furthermore, the absence of detailed dietary records represents a potential confounding factor known to influence gut microbiota composition, which may limit causal inference and should be addressed in future studies. Finally, inherent differences in gut anatomy, immune development, and host‐microbe co‐evolution between mice and humans limit direct translational extrapolation.

In summary, our findings shed new light on the critical period for host‒microbiota co‐development. They established a theoretical foundation for targeting the preconception microbiome to prevent developmentally programmed diseases and provided a conceptual framework for the next generation of preventive medicine.

## 4 Experimental Section

### 4.1 Animals and Experimental Design

Throughout the study, C57BL/6J mice were sourced from the Department of Laboratory Animal Science at Peking University Health Science Center in Beijing, China. These animals were housed in specific pathogen‐free (SPF) facilities under a 12‐h light/dark cycle (lights on 6:00‒18:00). Unless stated otherwise, mice had ad libitum access to autoclaved food (Xietong Shengwu, Jiangsu, China) and water. Upon arrival, mice were acclimatized for 1 week prior to any experimental procedures.

In the first experiment, 7‐week‐old female and 9‐week‐old male mice were acquired and were acclimatized for 1 week in SPF facilities. The female mice were randomly assigned to either controls (CON) or antibiotics (ABX) groups. To induce preconception gut microbiome perturbations, the ABX group received twice‐daily oral gavage of an antibiotic mixture containing neomycin (100 mg/kg; Innochem, Beijing, China), metronidazole (100 mg/kg; Aladdin, Shanghai, China), and vancomycin (50 mg/kg; Innochem, Beijing, China) in the morning and evening, along with ad libitum access to ampicillin (1 mg/mL; Aladdin, Shanghai, China) in drinking water [59]. The CON group received vehicle (sterile water) via oral gavage on the same schedule. This oral gavage regimen was administered for 1 week. In the subsequent week, female mice were mated with male mice at a 1:1 ratio. Upon observation of vaginal plugs, pregnant mice were transferred to new SPF cages, and the date was designated as embryonic day 0.5 (E0.5). Fecal samples were collected from the dams at E18.5. After delivery, offspring were reared on SPF bedding and remained housed with their dams during the lactation period to allow normal maternal care and feeding. At weaning (3 weeks postpartum), offspring were separated from dams and subsequently housed individually under SPF conditions. All offspring were provided with identical sterile chow and autoclaved water after weaning. This housing strategy was designed to minimize prolonged shared environmental exposure and coprophagic microbial transfer between dams and offspring. At weaning (3 weeks postpartum), a subset of offspring (both sexes included) was humanely euthanized for the collection of fecal and tissue samples. Considering the influence of estrogen, fecal, and tissue samples collection from 8‐week‐old mice was performed only on a portion of male mice. At 8 weeks postpartum, additional male offspring from the CON group were administered either sterile water (CON‐V) or 0.5% dextran sodium sulfate (DSS) solution (CON‐D) for 12 consecutive days, and offspring from the ABX group also received sterile water (ABX‐V) or 0.5% DSS solution (ABX‐D) for the same duration. Each offspring was treated as an independent biological replicate.

In the second experiment, 7‐week‐old male C57BL/6J mice were acquired and acclimatized for 1 week in SPF facilities. All mice were treated with a mixture of antibiotics (neomycin (100 mg/kg), metronidazole (100 mg/kg), vancomycin (50 mg/kg) and ampicillin (1 mg/mL)) for 1 week to establish a pseudo‐sterile state, followed by a 4‐day recovery period. After recovery, the mice were randomly divided into two groups: group 1 received fecal microbiota transplantation (FMT) from adult offspring of the CON group, while group 2 received FMT from adult offspring of the ABX group. After 14 days of microbial colonization, groups 1 were administered either sterile water (CFV) or DSS solution (CFD) for 12 consecutive days, and group 2 also received sterile water (AFV) or DSS solution (AFD) for the same duration.

All protocols were approved by the Animal Welfare and Ethics Committee of Peking University (Approval No. LA2022657).

### 4.2 FMT Procedures

To perform the FMT experiment, fecal samples from five adult offspring of the CON group and five adult offspring of the ABX group were collected, respectively, and mixed by combining an equal weight of each sample. In each group, 200 mg of mixed stool was suspended in 10 mL sterile phosphate‐buffered saline (PBS) containing 20% (v/v) glycerol. An aliquot of 200 µL of the suspension was given to each mouse via oral gavage, four times within 12 days.

### 4.3 Colitis Induced by DSS and Assessment of Disease Severity

To induce colitis, DSS (molecular weight 36–50 kDa, MP Biomedicals, CA) was dissolved in drinking water at a final concentration of 0.5% (w/v) and given ad libitum for 12 consecutive days. Mice were monitored daily to assess disease severity. The disease activity index (DAI) comprised body weight loss, stool consistency, and fecal blood, as determined by the hemoccult fecal occult blood test (Beckman coulter, CA), were assessed by an investigator who was blinded to the treatment conditions. The scoring system is detailed in Table S7.

### 4.4 Histopathology and Quantitative Analysis

Hematoxylin and eosin (H&E) staining was used to assess intestinal mucosal development. Tissues from the ileum and colon were fixed in 10% formaldehyde–PBS solution for 24 h, dehydrated, embedded in paraffin, and sectioned at 5 µm thickness. After deparaffinization and hydration, sections were stained with H&E (Sigma‐Aldrich), mounted with neutral gum (ZSGB‐BIO), and promptly imaged. Histopathological scoring was performed by a veterinary pathologist based on predefined criteria (Table S8).

Periodic Acid‐Schiff (PAS) staining was performed to evaluate goblet cells development in the colons of juvenile offspring. Paraffin‐embedded colon sections were deparaffinized and incubated in 1% periodic acid solution (Sigma‐Aldrich) for 10 min. Subsequently, sections were stained with Schiff's reagent (Sigma‐Aldrich) for 40 min and counterstained with Harris hematoxylin solution (Sigma‐Aldrich) for 25 min. After each step, sections were rinsed with PBS. Finally, sections were mounted with neutral gum and immediately imaged.

Alcian Blue‐Periodic Acid Schiff (AB‐PAS) staining was performed to evaluate colon development and inflammation in adult offspring. Paraffin‐embedded colon sections were deparaffinized and incubated in Alcian Blue solution (Sigma‐Aldrich) for 30 min. Subsequently, sections were stained with 1% periodic acid solution (Sigma‐Aldrich) for 10 min, Schiff's reagent (Sigma‐Aldrich) for 40 min, and then counterstained with Harris hematoxylin solution (Sigma‐Aldrich) for 25 min. After each step, sections were rinsed with PBS. Finally, sections were mounted with neutral gum and immediately imaged.

Whole‐slide scanning of H&E, PAS‐stained and AB‐PAS‐stained sections was performed using a NanoZoomer (Hamamatsu, Shizuoka, Japan). The length and cell count of 20 randomly selected villi or crypts in each animal section were measured, and ImageJ software (RRID:SCR_002285) was used to obtain quantitative data. Histological scoring was performed by investigators who was blinded to the treatment conditions.

### 4.5 Transmission Electron Microscopy (TEM) and Quantitative Analysis

For TEM, colon tissue samples measuring 3×3 mm were fixed in 4% paraformaldehyde for 24 h at 4°C, followed by fixation with 1% osmium tetroxide. The tissues were embedded in neutral resin and sectioned into thin slices. The sections were stained with uranyl acetate and lead citrate and stored under dry conditions for imaging purposes, which were promptly conducted. TEM images of ultrathin sections of the colon were acquired using TEM (JEOL, Tokyo, Japan) at 120 kV, and ImageJ software (RRID:SCR_002285) was used to obtain quantitative data. Quantitative image analysis was performed o by investigators blinded to the treatment conditions.

### 4.6 Quantitative Real‐Time Polymerase Chain Reaction (qRT‐PCR)

Total RNA was extracted from offspring's colons using the Tiangen RNA Extraction Kit (TIANGEN, Beijing, China). Following the determination of RNA concentration, cDNA amplification was performed using a cDNA synthesis kit (Absin, Shanghai, China). qPCR reactions were carried out using the QuantStudio 5 system (Thermo Fisher Scientific, MA) with Toyobo SYBR Green qPCR Mix (Toyobo, Osaka, Japan) and gene‐specific primers (Table S9). mRNA expression was normalized using *Gapdh* as the reference gene, and mRNA levels were calculated using the 2^‐ΔΔCt^ method.

### 4.7 Assessment of Inflammatory Factors

The MILLIPLEX Mouse High Sensitivity T Cell Magnetic Bead Panel kit (Millipore, MA) was used following the manufacturer's protocol. Briefly, the protein supernatant extracted from adult offspring's colon tissue was incubated overnight in a 96‐well plate embedded with microbeads. Subsequently, the plate was incubated with detection antibodies for 1 h, followed by Streptavidin Phycoerythrin incubation for 30 min. The values were read using a calibrated Luminex 200 system (Luminex Corporation, TX).

### 4.8 Colonic Transit Time

The colonic transit speed was evaluated using the latent bead test, conducted on the second day after the DSS challenge. Briefly, mice were fasted (with access to water) for 12 h. They were then lightly anesthetized using isoflurane (RWD, Guangdong, China), and a 2.5 mm spherical plastic bead coated with glycerol was gently inserted into the distal colon using a glass rod positioned 2 cm from the anus. Subsequently, the mice were individually placed in bedding‐free cages, and the time from bead insertion to expulsion was recorded. The experiment was repeated twice with a 6‐h interval, and the average of the two experiments was calculated as the bead expulsion latency.

### 4.9 Small Intestinal Transit Time

Small intestinal transit speed was evaluated using the carmine red transit method. Mice were administered 200 µL of carmine red solution (6% carmine red (Macklin, Shanghai, China) dissolved in 0.5% carboxymethylcellulose (Bide, Shanghai, China) water solution) via oral gavage. After 45 min, the mice were euthanized by cervical dislocation. The abdominal cavity was opened and the small intestine was carefully removed from the pylorus to the ileocecal junction, minimizing any stretching of the intestinal tract. The small intestine was laid out in a straight line, and the proportion of the distance traveled by the carmine red solution to the total length of the small intestine was measured to assess intestinal transit function.

### 4.10 Clinical Study Design and Questionnaire Collection

This case‐control study was conducted between July 2022 and December 2023. Eligible children with a confirmed diagnosis of PIBD were recruited from pediatric outpatient clinics and wards of Peking University Third Hospital and Children's Hospital Capital Institute of Pediatrics in China. Healthy controls were randomly selected from the same hospitals (children without PIBD or other gastrointestinal disorders).

The inclusion criteria were: (1) children aged <17 years; (2) PIBD diagnosis confirmed by physicians based on clinical, endoscopic, radiological, and histopathological criteria; and (3) mothers who completed the questionnaire and provided informed consent (children ≥8 years also provided assent). The exclusion criteria were: (1) children with severe physical or mental illnesses other than PIBD; (2) mothers with serious physical or mental illnesses during preconception, pregnancy, or lactation periods; and (3) mothers or children with a history of antibiotic, probiotic, or prebiotic use within 3 months prior to enrollment.

Trained investigators collected data from both cases and controls using standardized questionnaires, which included: demographics (age, sex, and living environment); maternal factors (medication use and medical history during preconception, pregnancy, and lactation); and early‐life exposures (feeding patterns and antibiotic use).

This clinical study was approved by the Institutional Review Board of Peking University Third Hospital (Approval No.2022‐652‐02). Written informed consent was obtained from all participating mothers, and children aged ≥8 years also providing their own assent.

### 4.11 Metagenomic Sequencing and Data Processing

Fecal samples were collected from mice at three developmental stages: dams (maternal, MA; CON, *n* = 8; ABX, *n* = 8), juvenile offspring (JU, 3 weeks old; CON, *n* = 7; ABX, *n* = 7), and adult offspring (AD, 8 weeks old; CON, *n* = 10; ABX, *n* = 12). Microbial DNA was extracted from all samples using the QIAamp PowerFecal Pro DNA Kit (Qiagen, NRW, Germany), and DNA concentration was quantified using a fluorometric method. DNA libraries were prepared using the NEBNext Ultra DNA Library Preparation Kit (NEB, MA). Briefly, DNA was fragmented to ∼350 bp by sonication, followed by end‐repair, A‐tailing, adaptor ligation, and PCR amplification. Paired‐end sequencing was performed on the Illumina NovaSeq 6000 platform by Novogene (Tianjin, China).

Raw sequencing data were quality‐controlled using FastQC (v0.11.8), and host‐derived reads were removed by mapping to the mouse reference genome using Bowtie2 (v2.5.1) [60]. The remaining high‐quality, non‐host reads were used for taxonomic and functional profiling. Species‐level taxonomic profiling was performed using MetaPhlAn4 (v4.0.6) with the mpa_vJun23_CHOCOPhlAnSGB_202403 marker gene database [61]. Phylogenetic tree construction was also conducted within MetaPhlAn4. Functional profiling was conducted using HUMAnN3 (v3.9) in UniRef90 mode [61]. To facilitate pathway‐level interpretation, the KO (KEGG Orthology) output from HUMAnN3 was further converted to KEGG Level 1, 2, and 3 pathway annotations using the R package microeco (v1.15.0) [62].

### 4.12 16S rRNA Amplicon Sequencing and Data Processing

Fecal samples were collected from children with IBD (IBD_CH, *n* = 18) and healthy control children (HC_CH, *n* = 18), as well as their mothers (IBD children's mothers, IBD_MA, *n* = 11; healthy control children's mothers, HC_MA, *n* = 18), using Stool Nucleic Acid Collection and Preservation Tubes containing DNA stabilization solution (Norgen, ON, Canada).Total microbial DNA was extracted using the QIAamp PowerFecal Pro DNA Kit (Qiagen, NRW, Germany), and DNA concentration was quantified using a Qubit fluorometer. The V3–V4 hypervariable regions of the bacterial 16S rRNA gene were amplified using primers 341F (5’‐CCTAYGGGRBGCASCAG‐3’) and 806R (5’‐GGACTACNNGGGTATCTAAT‐3’). PCR products were purified with magnetic beads, quantified, and pooled in equimolar concentrations. After verification, libraries were constructed and sequenced on the Illumina NovaSeq 6000 platform (Illumina, CA) by Novogene (Tianjin, China).

Raw reads were trimmed to remove barcodes and primers, merged using FLASH (v1.2.11) [63], quality‐filtered using fastp (v0.23.1) [64], and chimeras were removed by comparison with the SILVA database (v138.1). Denoising was conducted using the DADA2 plugin in QIIME2 to generate high‐resolution amplicon sequence variants (ASVs). Taxonomic assignment was performed using a Naive Bayes classifier trained on the SILVA 138.1 reference database. Phylogenetic tree construction was also conducted within the QIIME2 framework [65].

### 4.13 Untargeted Metabolomics Profiling and Data Processing

For animal experiments, cecal contents were collected at three developmental stages: maternal (MA; CON, *n* = 6; ABX, *n* = 6), juvenile (JU; CON, *n* = 7; ABX, *n* = 7), and adult (AD; CON, *n* = 7; ABX, *n* = 7). In the clinical study, fecal samples were obtained from children with IBD (IBD_CH, *n* = 18) and healthy control children (HC_CH, *n* = 18), as well as their mothers (IBD children's mothers, IBD_MA, *n* = 11; healthy control children's mothers, HC_MA, *n* = 18). All samples were analyzed using liquid chromatography coupled with tandem mass spectrometry (LC‐MS/MS). Each sample was split into two aliquots and analyzed under both positive and negative ionization modes using a Waters ACQUITY Premier HSS T3 column (1.8 µm, 2.1 mm × 100 mm). The mobile phases consisted of 0.1% formic acid in water (A) and 0.1% formic acid in acetonitrile (B), with a gradient elution profile: 5%‒20% B in 2 min, 20%‒60% in 3 min, 60%‒99% in 1 min (held for 1.5 min), followed by re‐equilibration to 5% B. The column temperature was maintained at 40°C, with a flow rate of 0.4 mL/min and an injection volume of 4 µL. Mass spectrometric data were acquired on a SCIEX TripleTOF 6600 system in information‐dependent acquisition (IDA) mode. Source parameters were as follows: ion source gas 1 (GAS1), 50 psi; ion source gas 2 (GAS2), 60 psi; curtain gas (CUR), 35 psi; source temperature, 550°C; and ion spray voltage, +5000 V or −4000 V for positive and negative modes, respectively. TOF‐MS scans were collected in the 50–1000 Da range, and MS/MS spectra were acquired with a collision energy of ± 30 eV and a collision energy spread of 15 eV.

Raw mass spectrometry data were first converted to the mzXML format using ProteoWizard. Peak detection, alignment, and retention time correction were performed using the XCMS. Peaks with missing values in more than 50% of the samples within any group were excluded. Remaining missing values were imputed using KNN. Signal intensity drift was corrected using SVR normalization. For metabolites detected in both positive and negative ionization modes, redundant features were consolidated by retaining the identity with the highest annotation confidence and the lowest coefficient of variation (CV).

### 4.14 α and β Diversity Analyses

In the animal study, microbial α diversity was calculated using the Shannon index at the species level, and functional α diversity was assessed based on KEGG Level 3 pathways. β diversity was first computed using Bray–Curtis dissimilarity for both taxonomic (species‐level) and functional (KEGG Level 3) profiles, and visualized via principal coordinates analysis (PCoA, Figure S2). Specifically, to mitigate potential biases associated with relative abundance‐based approaches, we performed new β‐diversity analyses that account for compositionality and sparsity [66]. Briefly, zeros were handled using a minimal non‐zero replacement strategy, followed by and the resulting abundance matrix was subjected to centered log‐ratio (CLR) transformation with the CoDaSeq R package [67]. β diversity was then calculated based on Aitchison distance (Euclidean distance in CLR‐transformed space) and visualized via PCoA.

In the clinical study, α diversity was assessed at the ASV level using the Shannon, Simpson, and Chao1 indices. β diversity was calculated using unweighted UniFrac dissimilarity and visualized by PCoA (Figure S15C). For consistency, the same CLR‐ Aitchison framework was applied to the clinical cohort data, following the analytical approach used in the animal study.

Group comparisons were conducted using the Wilcoxon rank‐sum test for α diversity and PERMANOVA for β diversity. All diversity metrics and visualizations were implemented using scripts from the Parallel‐Meta Suite [68].

### 4.15 Orthogonal Partial Least Squares Discriminant Analysis (OPLS‐DA)

OPLS‐DA was performed using the MetaboAnalystR package (v1.0.1) in R [69]. Prior to analysis, metabolite data were log2‐transformed to reduce skewness, stabilize variance, and improve the suitability for linear modeling. Variable Importance in Projection (VIP) scores generated from the OPLS‐DA model were used for subsequent identification of differential metabolites. Model validation was conducted using permutation test to assess the robustness and significance of class discrimination. The permutation test's Q^2^ reflects the predictive ability of the model; values closer to 1 indicate better performance, and models with Q^2^ > 0.5 are generally considered robust and reliable. In this study, models with Q^2^ > 0.5 were considered indicative of significant global metabolic differences between groups, while those with Q^2^ ≤ 0.5 were interpreted as lacking significant separation. This evaluation criterion was applied consistently across both animal and clinical datasets.

### 4.16 Identification of Differential Species and Functional Pathways across Three Developmental Stages

We identified differential microbial features—both in prevalence and abundance—across maternal (MA), juvenile (JU), and adult (AD) stages, comparing CON and ABX groups.

Prevalence‐based differential species: At the MA stage, species were considered differentially prevalent if detected in ≥50% of samples in one group and in ≤1 sample in the other. For JU and AD stages, where no direct antibiotic intervention occurred, thresholds were relaxed: species present in ≥30% of one group and in ≤1 sample of the other were considered differentially prevalent. Specifically for JU (*n* = 7 per group), to avoid marginal contrasts (e.g., 2 vs. 1), stricter criteria were applied: when a species was absent in one group, it had to appear in ≥2 samples in the other; if present in 1 sample, the opposite group required ≥3 samples for the species to qualify as differentially prevalent. Abundance‐based differential species: Species with significantly different relative abundances were identified using LEfSe [70]. For significance, the MA group used LDA > 2.0 and Wilcoxon *p* < 0.05; JU and AD groups used a more lenient threshold LDA > 2.0 and *p* < 0.1 to accommodate no direct antibiotic exposure.

Prevalence‐based differential pathways: Due to the inherently broader distribution and lower specificity of functional features compared to taxonomic ones, we applied relaxed prevalence thresholds. Pathways were deemed differentially prevalent if present in ≥25% of samples in one MA group or ≥15% in one JU/AD group, and in ≤1 sample of the opposite group. Specifically, mirroring the species‐level approach, when the observed prevalence was exactly at the threshold (e.g., 25% or 15%), we applied a stricter rule to minimize false positives, requiring no detection in the opposing group. When the prevalence exceeded the threshold, detection in up to one sample was permitted. Abundance‐based differential pathways: LEfSe analysis was also used for functional abundance differences. For the MA stage, thresholds were LDA >2.0 and *p* < 0.05; for JU and AD stages, LDA >2.0 and *p* < 0.1 were applied, consistent with the species‐level analysis rationale that no direct antibiotic exposure occurred during these stages.

### 4.17 Identification of Differential Metabolites across Three Developmental Stages

Differential metabolites were separately identified at three developmental stages (MA, JU, and AD) using orthogonal partial least squares discriminant analysis (OPLS‐DA), univariate statistical testing, and fold change thresholds. For the MA stage, metabolites were considered significantly different if they met the following criteria: a variable importance in projection (VIP) score >1, *p*‐value <0.05, and a fold change (FC) > 3/2 or < 2/3. For the JU and AD stages, which did not involve direct antibiotic exposure but reflected maternal preconception intervention, a slightly relaxed fold change threshold was applied. Specifically, in the JU stage, metabolites were considered differential if VIP >1, *p* < 0.05, and FC >4/3 or <3/4. For the AD stage, OPLS‐DA did not yield a robust model (permutation test Q^2^ < 0.5); therefore, VIP scores were not considered, and differential metabolites were defined solely by *p* < 0.05 and FC >4/3 or <3/4.

VIP scores were obtained from the OPLS‐DA as described above. For univariate analysis, statistical significance was assessed using Student's *t*‐test for normally distributed data or the Wilcoxon rank‐sum test for non‐normally distributed data. Fold change values were calculated as the ratio of metabolite levels in the ABX group relative to the CON group (ABX/CON).

### 4.18 Definition and Correlation Analysis of Intergenerationally Consistent Differential Features

Based on the criteria for identifying stage‐specific differential features—including species, functional pathways, and metabolites—we defined intergenerationally consistent differential features as those exhibiting significant differences between CON and ABX groups in at least two developmental stages with consistent directionality (e.g., enriched or more prevalent or upregulated in the same group). These features were further annotated using phylogenetic relationships for species, KEGG hierarchical levels for pathways, or chemical categories for metabolites, and visualized in composite circular heatmaps to illustrate their cross‐stage consistency.

Intergenerationally consistent microbe–metabolite correlation network analysis: Only samples with matched metagenomic and metabolomic data were included. Significant correlations were defined as those with |R| > 0.5 and *p* < 0.05, calculated using Spearman's rank correlation implemented in the Hmisc package (v5.2‒3) in R 4.4.1. Network visualization was performed using Cytoscape (v3.9.1) [71], where microbial nodes were colored orange‐pink and metabolite nodes blue. Edges represent significant correlations, with red indicating positive and blue indicating negative associations. Edge thickness and color depth correspond to the strength of the correlation (R). The betweenness centrality of each node was calculated and used to scale both node size and label font, highlighting the topologically influential features in the network.

Intergenerationally consistent features‐host phenotypes correlation network analysis: For each feature, we evaluated its association with both JU and AD phenotypes. Features showing concordant correlations with both (i.e., both positive or both negative) were colored green (positive) or red (negative); those with discordant correlations (one positive, one negative) were labeled blue; features with no significant correlation with either were shown in gray. Network density was computed as the ratio of observed edges to all possible edges, reflecting the compactness of the phenotype‐feature association network. An analogous analysis was conducted for differential metabolites.

### 4.19 Microbial Source Tracking Using FEAST

We employed the FEAST (Fast expectation–maximization for microbial Source Tracking) algorithm to estimate microbial source contributions across developmental stages [72]. In the animal study, microbial source tracking was performed separately for the CON and ABX groups. For each group, we calculated microbial contributions across three stage‐wise transitions: maternal to juvenile (MA→JU), maternal to adult (MA→AD), and juvenile to adult (JU→AD). To ensure statistical robustness, FEAST analysis was independently repeated ten times per group per transition. The average contribution values from the ten runs were visualized in triangular source‐sink plots, and differences between the CON and ABX groups were assessed using the Wilcoxon rank‐sum test followed by FDR correction. For the clinical cohort, microbial source tracking was conducted using a similar procedure, with mother–child pairs serving as natural source–sink configurations. Analysis was likewise repeated per comparison to obtain reliable estimates and support statistical inference. All FEAST analyses were conducted with 1000 iterations per run as recommended.

### 4.20 Strain‐Level Microbial Transmission Analysis

Strain‐level profiling was performed using StrainPhlAn4 [61] (version 4.0.6) to assess maternal–offspring strain sharing. Briefly, metagenomic reads were first taxonomically profiled using MetaPhlAn4 (version 4.0.6), and species‐specific marker gene alignments were retained for downstream strain‐level analysis. Consensus marker sequences were extracted from MetaPhlAn mapping results for each sample and each species‐level genome bin (SGB). For each selected SGB, reference marker gene databases were constructed, and strain‐level phylogenetic reconstruction was performed using StrainPhlAn with default parameters optimized for accurate phylogenetic inference (–phylogphlan_mode accurate). Phylogenetic trees were built based on single‐nucleotide variants across marker genes. Pairwise phylogenetic distances between samples were extracted from the resulting trees and normalized by total branch length to obtain normalized phylogenetic distances (nGD).

To examine strain‐level relatedness across different maternal–offspring pairings and treatment conditions, we compared pairwise nGD between maternal (MA) and adult offspring (AD) samples. Pairwise comparisons were classified into four groups based on treatment and pairing status: CON paired (true mother–offspring pairs), ABX paired, CON unpaired, and ABX unpaired. Unpaired comparisons were generated by randomly pairing maternal and offspring samples within the same treatment group. Normalized phylogenetic distances were log10‐transformed for visualization and statistical comparison, with lower log10(nGD) values indicating higher strain‐level similarity.

Strain‐sharing events were identified based on nGD using the strain_transmission.py script implemented in StrainPhlAn. For each SGB, strain‐level phylogenetic trees were analyzed together with sample metadata specifying maternal–offspring relationships and treatment groups. A strain sharing event was defined as a maternal–offspring sample pair with a nGD below the strain identity threshold. Following previous studies and recommended protocol, a threshold of 0.03 was applied [61, 67, 73]. Using this criterion, strain sharing events were identified for each SGB and summarized separately for CON and ABX groups.

### 4.21 Persisting Microbial Profiles across Developmental Stages

We constructed shared species networks across the three developmental stages in both CON and ABX groups. Specifically, for each group, we defined a species as “present” in a given stage if its relative abundance was greater than zero in at least one sample from that stage. For each group, the presence‐absence data of all species across the three stages were compiled to identify overlapping species subsets. Species shared by multiple stages were then visualized as nodes in a network, where edges were drawn between each species and the stages in which it was detected. For instance, a species present in all three stages (MA, JU, and AD) was connected to all three nodes, whereas a species found only in MA and JU was linked to these two nodes only. This visualization approach provided a network‐based representation, enabling intuitive interpretation of microbial persistence or stage‐specific presence patterns.

### 4.22 Microbial Colonization Patterns

We classified species into three colonization patterns based on prevalence differences between juvenile (JU) and adult (AD) stages: early colonization (higher prevalence in JU), persistent colonization (comparable prevalence between JU and AD), and late colonization (higher prevalence in AD). Prevalence was defined as the proportion of samples in which a species was detected (i.e., relative abundance > 0) at each stage. Only species detected in at least one sample of JU or AD were included. To assess the statistical significance of prevalence differences, a binomial test was applied for each species between JU and AD stages. Sample sizes were set according to the actual number of samples per stage. Species with a binomial test *p*‐value < 0.1 were considered to exhibit significant prevalence shifts and were assigned to colonization patterns based on the direction of the AD‐JU prevalence difference. To visualize the distribution of colonization patterns, volcano plots were generated using the difference in prevalence between AD and JU (AD‐JU) as the *x*‐axis and the negative log‐transformed *p*‐value as the *y*‐axis. Each point represents a species and is color‐coded by colonization type. The relative proportion of species in each colonization patterns was also annotated in the plot. Analyses were performed separately for CON and ABX groups.

### 4.23 Developmental Trajectory

Building on the observed differences in colonization patterns between the CON and ABX groups, we further conducted species‐level comparisons of microbial developmental trajectories. Only species that met the inclusion criteria for colonization pattern analysis in both groups were included, ensuring biological relevance and minimizing statistical bias from low‐prevalence taxa. For each included species, we calculated the change in prevalence from JU to AD (AD−JU) within both the ABX and CON groups. Each species was then represented as a point in a 2D coordinate system, with the *x*‐axis indicating the change in the ABX group and the *y*‐axis indicating the change in the CON group. The position of each point reflects how the species' developmental trajectories differ between the two groups. To assess overall differences in developmental trajectories between groups, we performed a paired Wilcoxon test on all coordinate pairs. A significant result (*p* < 0.05) indicates that maternal preconception antibiotic exposure broadly disrupts the developmental progression of gut microbiota.

Theoretically, if developmental trajectories are consistent across groups, all points should align along the diagonal (*y* = *x*). However, in our analysis, the majority of points were located above the diagonal (*y* > *x*), indicating that the prevalence change from JU to AD (AD‐JU) was generally greater in the CON group than in the ABX group. For example, consider a point located above the diagonal (e.g., with coordinates (0, 50)), where the *x*‐axis represents the AD–JU prevalence difference in the ABX group, and the *y*‐axis represents that in the CON group. This means that in the ABX group, the species shows no significant prevalence change between JU and AD stages (AD–JU = 0), indicating a “persistent colonization” pattern. In contrast, in the CON group, the same species increases markedly in prevalence from JU to AD (AD–JU = 50), corresponding to a “late colonization” pattern. This suggests that microbial developmental trajectory was relatively accelerated in the ABX group, as several taxa had already reached adult‐like prevalence by the juvenile stage—while in the CON group, the same taxa continued to increase prevalence from juvenile to adult. To enhance visual interpretation, each point was colored by phylum‐level taxonomy, and sized according to the absolute difference in prevalence changes between groups. A reference diagonal (*y* = *x*) was also included to aid in identifying the direction and extent of trajectory deviations.

### 4.24 Partitioning of β Diversity Into Turnover and Nestedness

To investigate the compositional structure of microbial communities, we partitioned total β diversity into species turnover (replacement) and nestedness components following Baselga [46]. This approach is based on presence–absence data to focus on compositional differences. Pairwise β diversity was calculated using the beta.pair() function from the betapart R package (v1.6.1), which yields three matrices: total β diversity (β_sor), turnover (β_sim), and nestedness (β_sne). For each sample pair, the proportion of turnover was computed as β_sim/β_sor, and the nestedness proportion as 1 − turnover proportion. For comparisons between treatment groups within each developmental stage (CON_MA vs. ABX_MA, CON_JU vs. ABX_JU, CON_AD vs. ABX_AD), we calculated the mean turnover and nestedness proportions across all relevant sample pairs. Statistical significance of differences in turnover proportion was assessed using a permutation test (999 random shuffles of group labels), with the observed mean turnover compared against the null distribution to obtain empirical *p*‐values. All analyses were conducted in R (v4.4.1).

### 4.25 Ecological Niche Breadth

Ecological niche breadth was primarily calculated based on species abundance using Levins index, implemented with the niche.width function in the R package spaa (method = “levins”), and additionally assessed using the Shannon–Wiener index as a complementary metric (method = “shannon”) to evaluate robustness. To account for the potential biases from detection limits, species counts were rarefied to the minimum sequencing depth before recalculating niche breadth. Across both metrics and data representations (abundance or rarefied counts), consistent trends were observed, ensuring the robustness of the results (Figures S12 and S18). Wilcoxon rank‐sum tests were performed to evaluate differences in niche breadth across stages.

### 4.26 Community Assembly Processes Based on Null Model

We first calculated pairwise βNTI using observed and null distributions of β‐mean nearest taxon distance (βMNTD), based on a reference phylogenetic tree and species‐level (in animal, metagenome data) or ASV‐level (in human, 16s data) abundance profiles. Null communities were generated through taxa label shuffling across the phylogeny (*n* = 1000 permutations). To complement phylogenetic inference, we further calculated RC_Bray_ by comparing observed Bray‒Curtis dissimilarities with those from null communities constructed via abundance‐weighted constrained randomization (*n* = 500 permutations), preserving observed species richness and total sample abundance. Because this null‐model framework relies on count‐based abundance information, all samples were rarefied to the minimum sequencing depth prior to analysis. βNTI and RC_Bray_ values were then used to infer dominant ecological processes as follows [47]: Heterogeneous selection: βNTI > +2; Homogeneous selection: βNTI < –2; Dispersal limitation: |βNTI| ≤ 2 and RC_Bray_ > +0.95; Homogenizing dispersal: |βNTI| ≤ 2 and RC_Bray_ < –0.95; Undominated processes: |βNTI| ≤ 2 and |RC_Bray_| ≤ 0.95. The relative contributions of ecological processes were visualized using stacked bar plots.

In animal, we first assessed community assembly processes within each developmental stage (i.e., CON_MA, ABX_MA, CON_JU, ABX_JU, CON_AD, ABX_AD), followed by across developmental stage (i.e., CON_MA ‐> CON_JU, ABX_MA ‐> ABX_JU; CON_MA ‐> CON_AD, ABX_MA ‐> ABX_AD; CON_JU ‐> CON_AD, ABX_JU ‐> ABX_AD). In human, due to the cross‐sectional design in which child–mother samples were collected simultaneously rather than longitudinally, the data did not represent true developmental transitions. Therefore, we only assessed community assembly processes within each group.

### 4.27 Community Assembly Processes Based on Neutral Community Model

We applied the neutral community model (NCM) to estimate the extent to which taxa dynamics could be explained by neutral processes [74]. Because this analyze relies on count‐based abundance information, all samples were rarefied to the minimum sequencing depth prior to analysis. For each group, the model was fitted based on the relationship between the mean relative abundance of each taxon and its frequency of occurrence across samples. The model was parameterized using nonlinear least squares to estimate the migration rate (m), with confidence intervals and goodness‐of‐fit (R^2^) computed to assess model performance. *R* ^2^ quantifies the degree to which the observed data conform to the neutral expectation. Higher *R* ^2^ values indicate that microbial community assembly is more consistent with stochastic processes, whereas negative *R* ^2^ values suggest that the neutral model fails to capture the observed patterns, implying that deterministic forces (e.g., environmental selection) predominantly shape community structure. Nm (the migration number), calculated as the product of metacommunity size (N) and migration rate (m), reflects the overall dispersal capacity. Higher Nm values denote more effective microbial dispersal and more homogeneous taxonomic distribution across samples.

In the animal experiment, we first fitted the NCM for each developmental group individually (i.e., CON_MA, ABX_MA, CON_JU, ABX_JU, CON_AD, ABX_AD), followed by across stages (i.e., CON_MA ‐> CON_JU, ABX_MA ‐> ABX_JU; CON_MA ‐> CON_AD, ABX_MA ‐> ABX_AD; CON_JU ‐> CON_AD, ABX_JU ‐> ABX_AD). In the human cohort, also due to the cross‐sectional sampling design, we only assessed the neutral model within each group separately.

### 4.28 Dispersal‐Shifted Taxa and Network‐Based Correlation Analysis

In each group, taxa were categorized into three dispersal states based on their deviation from model predictions: above prediction (higher observed occurrence than expected), neutral, and below prediction (lower than expected). Taxa were considered to have higher dispersal in ABX_AD if their dispersal state was elevated compared to their counterpart in CON_AD (e.g., “above prediction” in ABX_AD versus “neutral” or “below prediction” in CON_AD). Conversely, taxa were classified as having lower dispersal in ABX_AD if their state was lower than in CON_AD.

To investigate potential ecological interactions involving these dispersal‐shifted taxa, we constructed co‐occurrence networks within the ABX_AD group. Specifically, Spearman correlations were computed between the high‐ and low‐dispersal taxa and all other taxa. Edges were retained only if |r| > 0.8 and *p* < 0.05, providing a stringent threshold for robust associations. Edge color and thickness reflected the strength and direction of correlation: red for positive and blue for negative correlations, with deeper color and thicker lines indicating stronger correlations. Despite the strict threshold applied, a large number of significant associations were still observed; therefore, positive and negative correlations were visualized separately to improve clarity. Importantly, the spatial positioning of taxa nodes remained consistent across both network panels.

### 4.29 Partial Least Squares Path Modeling

We applied partial least squares path modeling (PLS‐PM) using the R package plspm. This model was designed to quantify the effects of maternal exposure on the adult microbial community, mediated by key ecological processes. The latent variable Microbial Transmission was represented by the proportional contribution of the maternal (MA) microbiota to the adult offspring (AD) community, as estimated by FEAST. This variable reflects the degree to which maternal microbes are retained in the adult offspring, and thus a higher value in the ABX group indicates increased maternal‐to‐offspring microbial transfer under antibiotic exposure. The latent variable Developmental Trajectory was characterized using two manifest variables derived from the AD microbial community: the proportion of taxa showing persistent colonization patterns, and 1 minus the proportion of late colonization patterns’ taxa. This transformation allowed us to capture the concept of accelerated microbial maturation in ABX offspring, as it reflects both an enrichment of persistent‐colonizing species and a depletion of late colonizers. Community Assembly was modeled using the β‐nearest taxon index (βNTI) between MA and AD communities and the proportion of low‐dispersal taxa in AD, both of which are indicative of more deterministic and selective community assembly processes. Finally, the adult offspring microbial community was described by both α and β diversity metrics, including Shannon and Simpson indices as indicators of within‐sample diversity, and the principal coordinates in PCoA to reflect between‐sample variation.

### 4.30 Identification of Differential Microbial and Metabolic Signatures in the Clinical Cohort

Given our animal study findings that intergenerational microbial features tend to be more stable and conserved—often showing consistent differences across both generations and stronger correlations with host phenotypes—the same stringent criteria were applied to both mothers and children when identifying differential taxa. Differential microbial taxa were identified using LEfSe analysis, with LDA scores >2 and *p*‐values <0.05 were considered significant and visualized using lollipop plots. Differential metabolites were screened using group‐specific criteria: for children (CH), metabolites with VIP >1, *p* < 0.05, and fold change (FC) >3/2 or <2/3 were selected; for mothers (MA), only *p* < 0.05 and FC >4/3 or <3/4 were applied. VIP was not considered in mothers due to the lack of clear separation in OPLS‐DA, and the FC threshold was relaxed accordingly, as all children's mothers appeared healthy.

### 4.31 Multivariable Association Analysis Using MaAsLin2

To account for potential confounding factors in clinical microbiome data, we performed multivariable association analysis using MaAsLin2 [75] (v1.20.0) in R. For children, associations between microbial features and disease status (PIBD vs. HC) were evaluated while adjusting for age, gender, delivery mode, and feeding patterns. For mothers, only delivery mode and feeding patterns were included as covariates due to limited demographic information. Input data consisted of CLR‐transformed microbial abundances, and the analysis method was linear modeling. Differential species were identified using thresholds consistent with those applied in the LEfSe analyses: *p* < 0.05 for children and mother samples.

### 4.32 Mantel Test for Microbiome–Metabolome Associations

To explore the correlation between microbial and metabolic alterations in children and mothers, we performed a Mantel test to assess the association between the overall dissimilarity matrices of differential ASVs and differential metabolites. Spearman correlations were calculated for the differential microbial correlation. To visualize these associations, a correlation matrix was plotted, with each cell representing the pairwise Spearman correlation between differential ASVs. The color and size of each cell reflected the correlation strength and direction—larger, darker cells indicating stronger correlations, with red and blue denoting positive and negative associations, respectively. Furthermore, metabolites were categorized into three groups: (1) Upregulated metabolites, (2) Downregulated metabolites, and (3) Metabolites consistently altered in both mothers and children. Mantel tests were conducted between each individual metabolite group and each differential ASV. The results were visualized using network‐style connections, where colored lines connected metabolites to microbial species with significant Mantel associations. The color of the connecting lines represented statistical significance: red for *p* < 0.01, green for 0.01 ≤ *p* < 0.05, and line thickness corresponding to the Mantel correlation coefficient.

### 4.33 Differential Signatures Correlation Analyses Between Children and Mothers

To explore the potential intergenerational relationships of microbial and metabolic alterations, we assessed the correlations between differential features identified in mothers and their children. Two complementary approaches were used: Procrustes analysis to evaluate overall concordance, and redundancy analysis (RDA) to identify maternal drivers of child‐specific variation.

For microbial profiles, Procrustes analysis was performed using the set of significantly different taxa identified in children and in mothers, respectively. The analysis revealed a significant alignment between the two datasets (*p* < 0.05), suggesting a non‐random correlation between maternal and child microbial alterations. Redundancy analysis (RDA) was subsequently conducted to determine which maternal differential taxa could explain the variance in the child differential microbiome, highlighting candidate microbes potentially involved in the shaping of the child's microbial landscape. Similarly, for metabolic features, Procrustes analysis was conducted using all significantly altered metabolites from children and mothers. A significant correspondence was also observed between the two groups, suggesting a transgenerational metabolic association. To further clarify the potential maternal drivers of child metabolic divergence, RDA was performed using only the differential metabolites that were consistently altered in both mothers and children, allowing for a more focused and interpretable visualization of maternal influence on offspring metabolic patterns.

### 4.34 Identification of Antibiotic Mother‒Child Shared Microbes and Machine Learning

We first identified shared ASVs between mothers and their children, as previously described. Network diagrams were used to visualize shared and unique microbial members. To further identify key discriminative features, we applied random forest (RF) analysis, selecting variables with mean decrease in accuracy > 0 as important microbial predictors. Based on these selected features, we constructed classification models using five representative machine learning algorithms: Linear Discriminant Analysis (LDA), K‐Nearest Neighbors (KNN), Naive Bayes (NB), Random Forest (RF), and eXtreme Gradient Boosting (XGBoost). Model performance was evaluated using a combination of stratified fivefold cross‐validation (CV) and bootstrap validation: Stratified fivefold CV was used to preserve class proportions while partitioning the data (training: test = 4:1), repeated five times to mitigate partition bias. Bootstrap validation was performed with 1000 iterations of resampling with replacement, generating an empirical distribution of AUC scores. Receiver Operating Characteristic (ROC) curve and Area Under the Curve (AUC), computed using the trapezoidal rule. 95% confidence intervals for AUC were estimated from bootstrap distributions. Mean Squared Error (MSE) and F1‐score were also calculated to assess prediction accuracy and balance between precision and recall. The solutions for overfitting issues encountered with small samples in this work are as follows: (1) For LDA, enable regularization and adjust the shrinkage intensity. (2) For KNN, increase the number of neighbors, increase the leaf size, and use distance weighting. (3) For Naïve Bayes, reduce the smoothing value. (4) For Random Forest, control the number of trees, limit the depth of the trees, increase the minimum number of samples required to split a node, increase the minimum number of samples per leaf, and perform random selection during each tree split. (5) For XGBoost, limit the number of trees, limit the tree depth, use a low learning rate, reduce the proportion of random sampling of samples for each tree, reduce the proportion of random sampling of features for each tree, decrease L1 regularization, and increase L2 regularization.

All analyses were conducted using Python 3.9. Key libraries and functions included scikit‐learn (1.2.2) for LDA, KNN, NB, RF, xgboost (1.7.5) for XGBoost, sklearn.model_selection for StratifiedKFold, sklearn.metrics for ROC curve, AUC, bootstrap, and percentile for confidence interval.

### 4.35 Statistical Analysis

Animal Study: The data are expressed as the mean ± standard error of the mean (SEM). Normality was assessed using Shapiro–Wilk and Kolmogorov–Smirnov tests. For comparisons between two groups, unpaired two‐tailed Student's *t*‐test (parametric data) or Mann–Whitney *U* test (non‐parametric data) were applied. For comparisons among more than two groups, one‐way analysis of variance (ANOVA) followed by Tukey's multiple comparison test or non‐parametric tests (Kruskal–Wallis test followed by Dunn's multiple comparison test) were performed. When involving two variables, two‐way ANOVA was used for analyses, followed by Tukey's or Sidak's multiple comparison test. The specific statistical tests used for each panel are described in the figure legends. The sample size (*n* value) differed across experiments and is specified in each figure legend. The bar graphs represent individual biological replicates as single points. Statistical significance was set at *p* < 0.05. Statistical analyses were performed using GraphPad Prism 10 software (GraphPad Software, CA).

Clinical Study: Continuous variables (age) were reported as mean ± standard deviation (SD), and the independent samples *t*‐test was used to compare the two groups. Cohen's d was calculated, with values of 0.2, 0.5, and 0.8 generally interpreted as small, medium, and large effects, respectively. Categorical variables were analyzed in terms of number and percentage (n, %), and the Chi‐square test was used to compare the two groups. All statistical analyses were performed using SPSS software version 26.0 (SPSS Inc., IL). For categorical variables, odds ratios (OR) with 95% confidence intervals (CI) were provided.

## Author Contributions

Liping Duan, Yuzhu Chen, and Cunzheng Zhang formulated the scientific question. Yuzhu Chen designed the study and carried out all bioinformatics analyses for both the animal and clinical study. Ruqiao Duan and Cunzheng Zhang conducted the animal experimental and collected clinical samples. Gaonan Li, Xiaolin Ji, and Qi Zhang provided assistance with the animal experimental. Fei Pei and Kun Wang aided in image acquisition and analysis. Yuzhu Chen and Ruqiao Duan drafted the manuscript. Liping Duan oversaw the study and performed critical revisions of the manuscript. All authors provided comments on the paper and approved the final manuscript.

## Conflicts of Interest

The authors declare no conflicts of interest.

## Supporting information




**Supporting File 1**: advs74788‐sup‐0001‐FigureS1‐S18.docx.

**Supporting File 2**: advs74788‐sup‐0002‐TableS1‐S18‐.xlsx.

## Data Availability

16S rRNA and metagenomic sequencing data were submitted to the NCBI Sequence Read Archive (SRA) database under the study accession numbers PRJNA1290863, PRJNA1288143. Untargeted metabolomic data were submitted to the NGDC OMIX database under study accession numbers PRJCA043150, PRJCA043138.
