# Supplementary material for: Maternal Preconception Antibiotic Exposure Disrupts Microbial Succession: A Transgenerational Risk for Offspring Gut Mucosal Immaturity and Colitis Susceptibility
Source: Adv Sci (Weinh). 2026 Apr 2;13(29):e16931. doi: 10.1002/advs.202516931 (PMC13205903; doi:10.1002/advs.202516931)
Supplement: Supplementary file 1 — Supporting File 1: advs74788‐sup‐0001‐FigureS1‐S18.docx. [file ADVS-13-e16931-s001.docx]

**Supporting Information**

**Maternal Preconception Antibiotic Exposure Disrupts Microbial Succession: A Transgenerational Risk for Offspring Gut Mucosal Immaturity and Colitis Susceptibility**

Yuzhu Chen,^1,2,5^ Ruqiao Duan,^1,2,5^ Cunzheng Zhang,^1,3,5^ Gaonan Li,^1,2^ XiaoLin Ji,^1,2^ Qi Zhang,^1,2^ Fei Pei,^4^ Kun Wang,^1,2^ Liping Duan^1,2,*^

^1^ Department of Gastroenterology, Peking University Third Hospital, Beijing 100191, China

^2^ PKUMed‐Wisbiom Joint Laboratory for Human Microbiome Research, Beijing, 100191, China

^3^ Department of Gastroenterology, The Affiliated Suzhou Hospital of Nanjing Medical University, Suzhou Municipal Hospital, Gusu School, Nanjing Medical University, Jiangsu, 215002, China

^4^ Department of Pathology, Peking University Third Hospital, Beijing 100191, China

^5^ These authors contributed equally to this work

^*^ Correspondence: duanlp@bjmu.edu.cn

**Supplemental Figure and Legend**

**
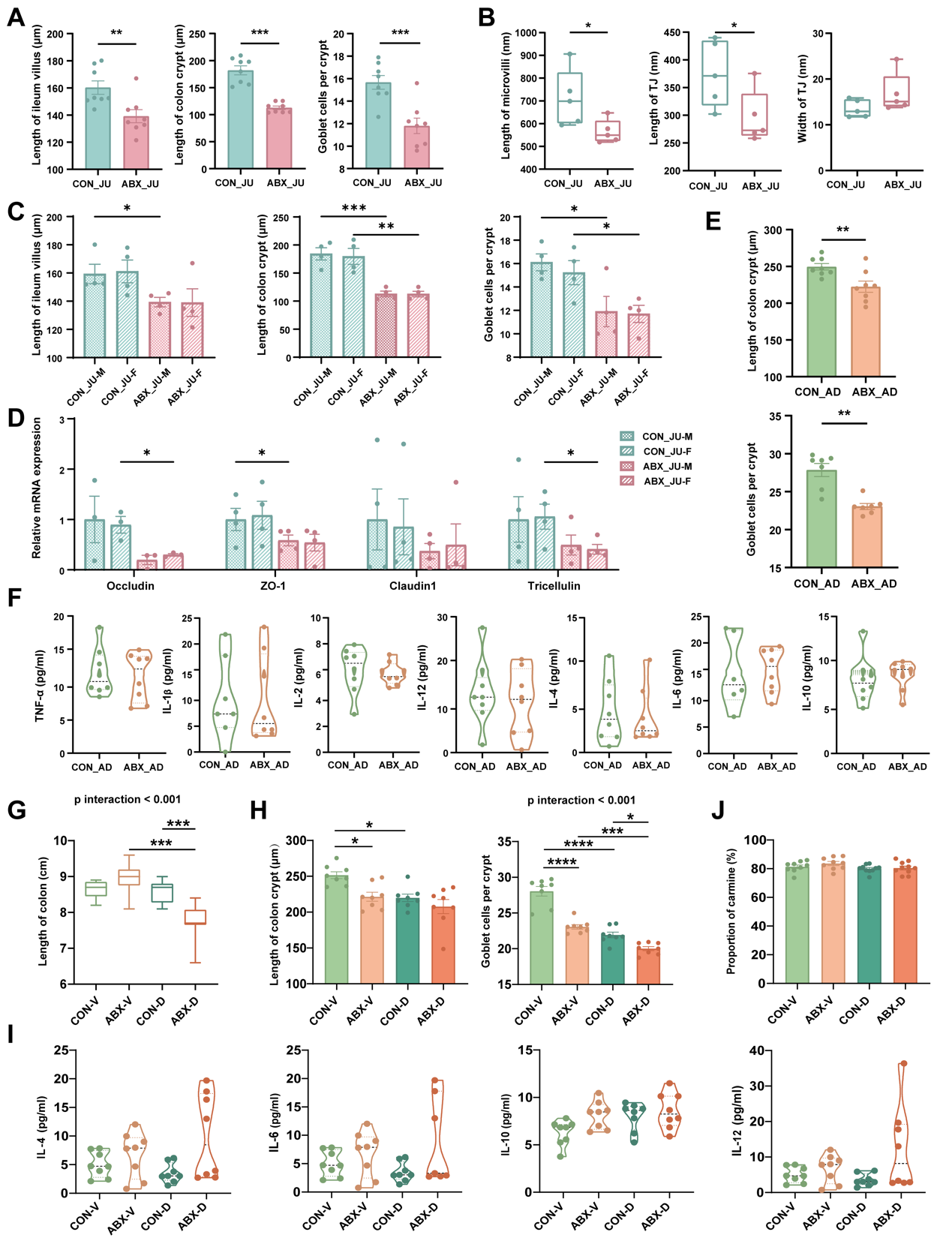
**

**Figure S1. Maternal preconception antibiotic exposure impairs the intestinal development and increases DSS-induced colitis susceptibility in offspring.**

(A) Villi length in the ileum, crypt depth in the colon, and goblet cells in the colonic crypt of juvenile offspring using hematoxylin and eosin (H&E) or Periodic-acid Schiff (PAS) staining (n = 8). (B) Measurement of colonic microvilli length, tight junctions (TJs) length and width between colonic epithelial cells in juvenile offspring (n = 5). (C) Villi length in the ileum, crypt depth in the colon, and goblet cells in the colonic crypt of male juvenile offspring (JU-M) and female juvenile offspring (JU-F) using H&E or PAS staining (n = 4). (D) Relative mRNA expression of colonic barrier-related genes (Occludin, ZO-1, Claudin1 and Tricellulin) in juvenile offspring of different sexes (n = 3-4). (E) Crypt depth in the colon and goblet cells in the colonic crypt of adult offspring using H&E or Alcian Blue-Periodic Acid Schiff (AB-PAS) staining (n = 8). (F) Concentrations of inflammatory factors (n = 6-8). (G) Length of colon (n=8). (H) Crypt depth in the colon and goblet cells in the colonic crypt of adult offspring using H&E or Alcian Blue-Periodic Acid Schiff (AB-PAS) staining (n = 8). (I) Concentrations of inflammatory factors (IL-4, IL-6, IL-10, IL-12) (n = 8). (J) Small intestine transit time (n = 8). Data are presented as mean ± SEM. Unpaired Student's *t*-test (A, B, E, F) was used for comparisons between two groups. One-way ANOVA followed by Tukey's multiple comparison test (C) and Kruskal-Wallis test followed by Dunn's multiple comparison test (D) were used for comparisons among four groups. Two-way ANOVA followed by Tukey’s multiple comparison test was used for analyses involving two variables (G, H, I, J). **P* < 0.05, ***P* < 0.01, ****P* < 0.001, *****P* < 0.0001.


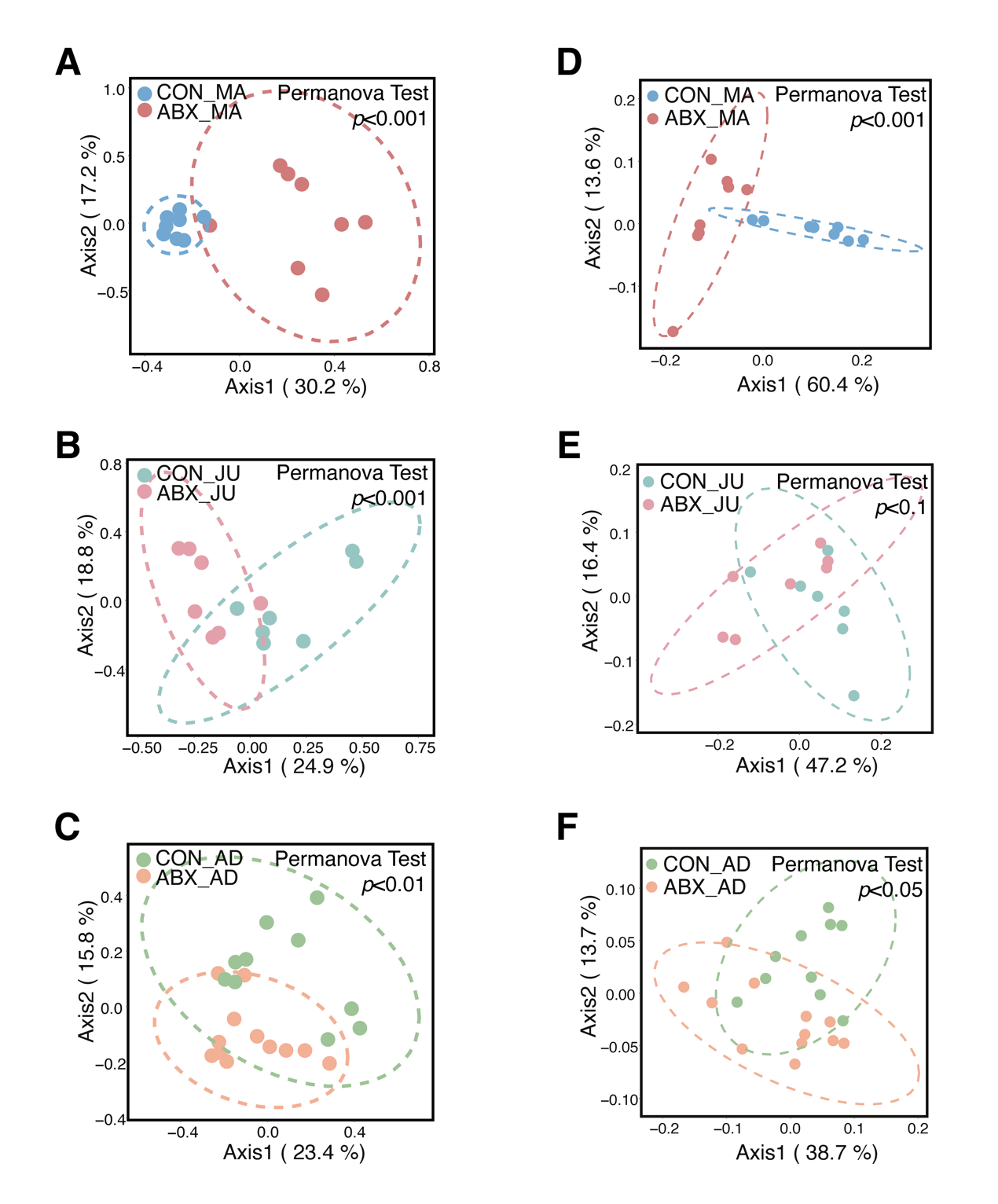


**Figure S2. Beta diversity of gut microbiome at different developmental stages based on Bray–Curtis distance after maternal preconception antibiotic exposure.**

(A) Maternal stage (MA) gut microbiome beta diversity at the species level (n = 8). (B) Juvenile stage (JU) beta diversity at the species level (n = 7). (C) Adult stage (AD) beta diversity at the species level (n = 10–12). (D) Maternal stage beta diversity at KEGG Level 3 functional pathways. (E) Juvenile stage beta diversity at KEGG Level 3 functional pathways. (F) Adult stage beta diversity at KEGG Level 3 functional pathways. Beta diversity differences were evaluated using the Permanova Test.


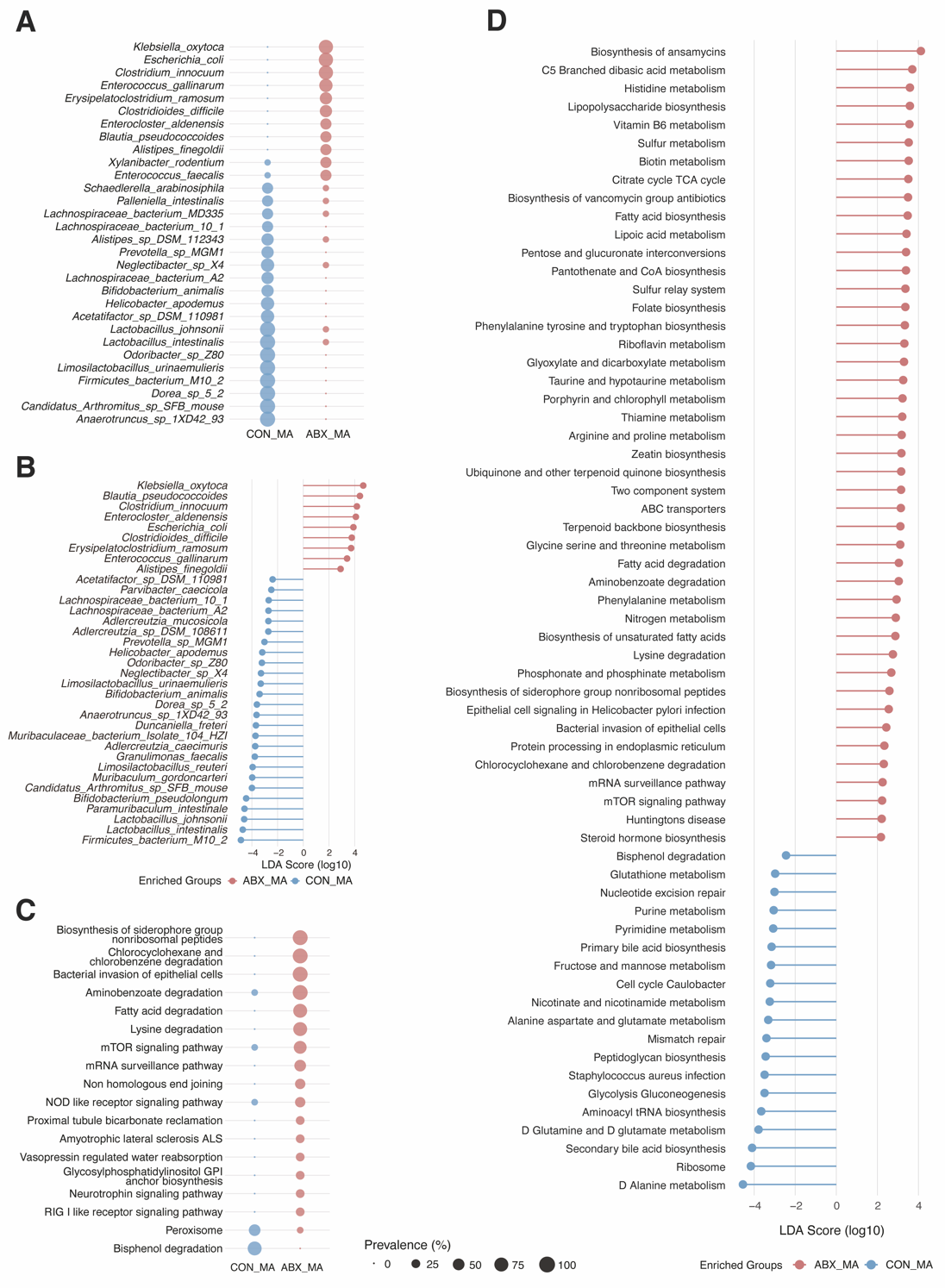


**Figure S3. Alterations of microbial and functional profiles at the maternal stage after maternal preconception antibiotic exposure.**

(A) Species with different prevalence between CON_MA and ABX_MA groups; circle size reflects species prevalence within each group. (B) Differentially abundant species between CON_MA and ABX_MA identified by LEfSe analysis (LDA score > 2, Wilcoxon test p < 0.05). (C) KEGG Level 3 pathways with prevalence differences between CON_MA and ABX_MA groups. (D) Differentially abundant KEGG Level 3 pathways between CON_MA and ABX_MA identified by LEfSe (LDA score > 2, Wilcoxon test p < 0.05). Sample size n=8.


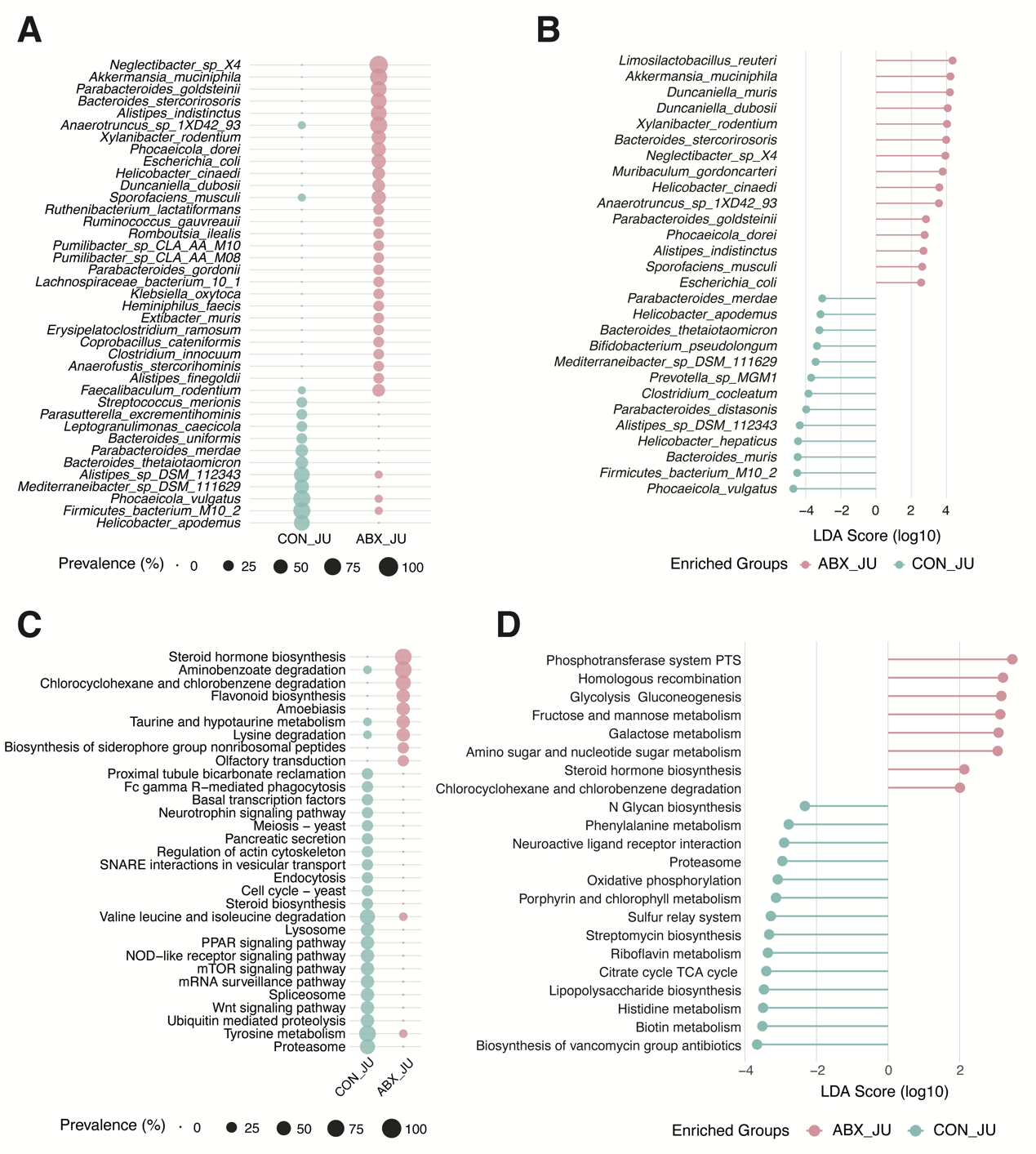


**Figure S4. Alterations of microbial and functional profiles at the juvenile stage after maternal preconception antibiotic exposure.**

(A) Species with different prevalence between CON_JU and ABX_JU groups; circle size reflects species prevalence within each group. (B) Differentially abundant species between CON_JU and ABX_JU identified by LEfSe analysis (LDA score > 2, Wilcoxon test p < 0.05). (C) KEGG Level 3 pathways with prevalence differences between CON_JU and ABX_JU groups. (D) Differentially abundant KEGG Level 3 pathways between CON_JU and ABX_JU identified by LEfSe (LDA score > 2, Wilcoxon test p < 0.05). Sample size n=7.


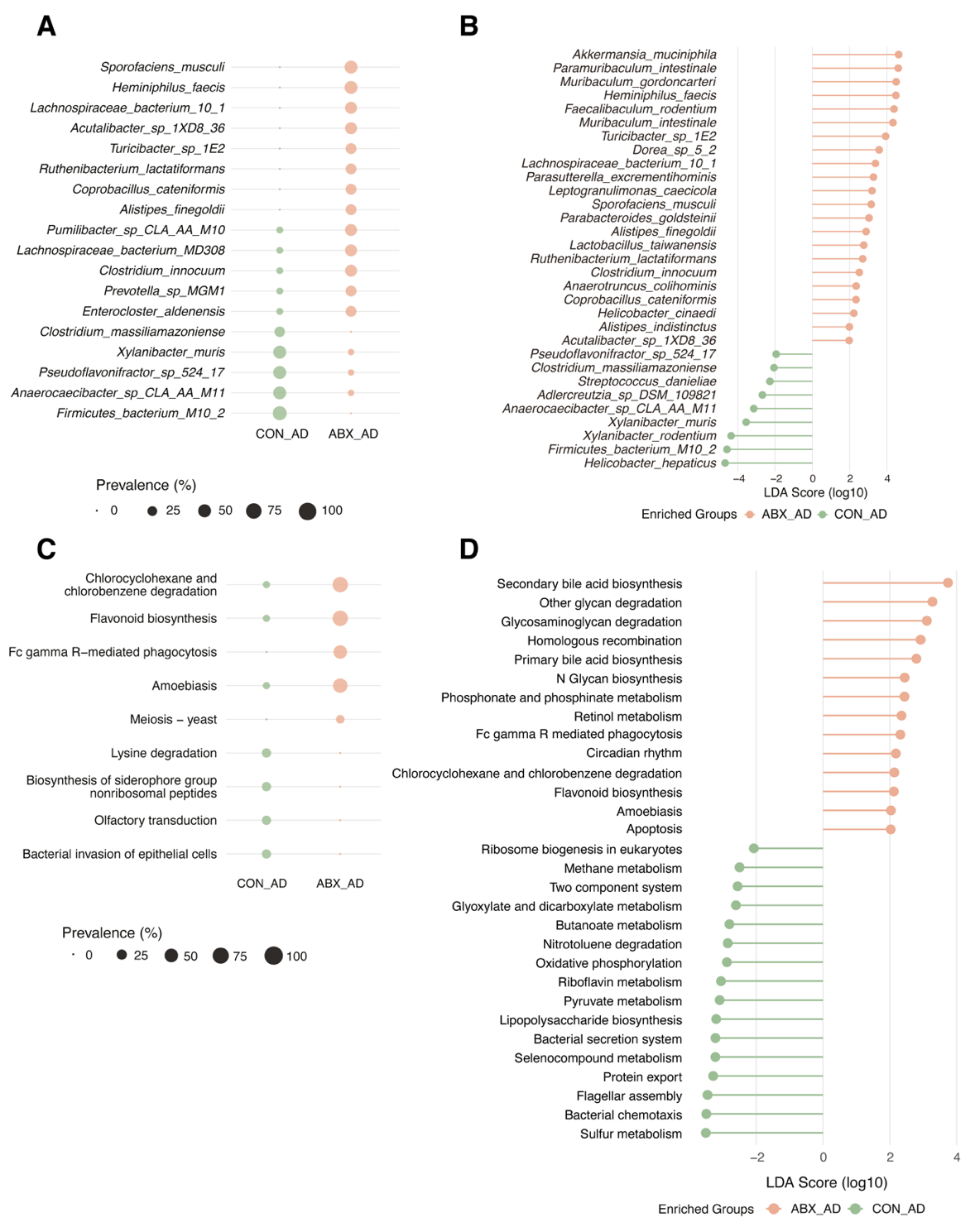


**Figure S5. Alterations of microbial and functional profiles at the adult stage after maternal preconception antibiotic exposure.**

(A) Species with different prevalence between CON_AD and ABX_AD groups; circle size reflects species prevalence within each group. (B) Differentially abundant species between CON_AD and ABX_AD identified by LEfSe analysis (LDA score > 2, Wilcoxon test p < 0.05). (C) KEGG Level 3 pathways with prevalence differences between CON_AD and ABX_AD groups. (D) Differentially abundant KEGG Level 3 pathways between CON_AD and ABX_AD identified by LEfSe (LDA score > 2, Wilcoxon test p < 0.05). Sample size n=10, 12.


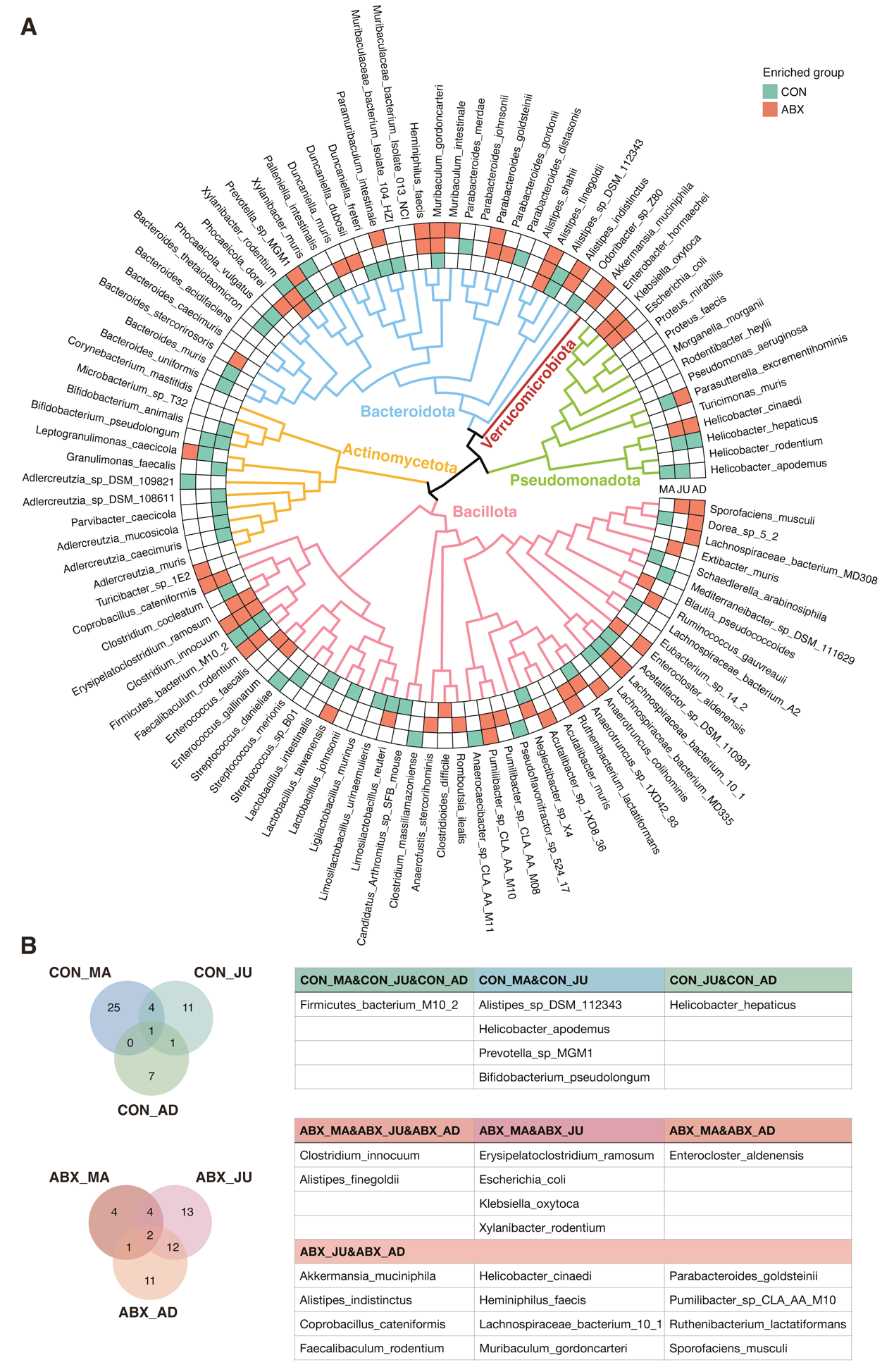


**Figure S6. Identification of consistent differential species across developmental stages following maternal preconception antibiotic exposure.**

(A) Circular heatmap with phylogenetic tree displaying species with significant enrichment or prevalence differences between CON and ABX groups across the maternal (MA), juvenile (JU), and adult (AD) stages. (B) Venn diagrams showing the overlap of significantly enriched or high-prevalence species across the three developmental stages (MA, JU, AD). Species that are differentially enriched in at least two stages with consistent direction (either CON or ABX) were defined as conserved differential species. Table summarizing conserved differential species, indicating the enriched group (CON or ABX) and the specific stages where enrichment was observed.


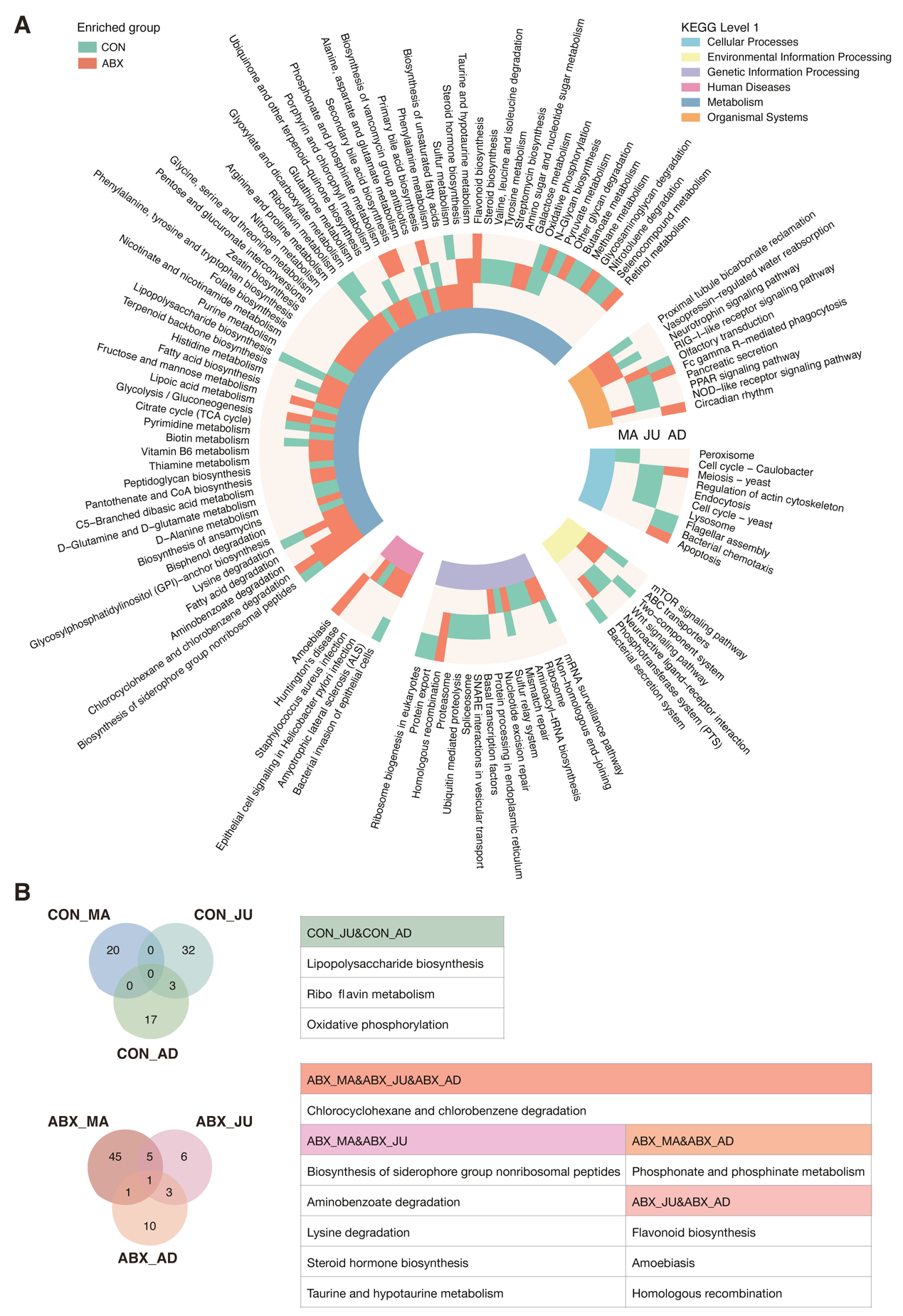


**Figure S7. Identification of consistent differential functional pathways across developmental stages following maternal preconception antibiotic exposure.**

(A) Circular heatmap with hierarchical functional classification displaying KEGG Level 3 pathways with significant enrichment or prevalence differences between CON and ABX groups across the maternal (MA), juvenile (JU), and adult (AD) stages. (B) Venn diagrams showing the overlap of significantly enriched or high-prevalence KEGG pathways across the three developmental stages (MA, JU, AD). Pathways that are differentially enriched in at least two stages with consistent direction (either CON or ABX) were defined as conserved differential pathways. Table summarizing conserved differential pathways, indicating the enriched group (CON or ABX) and the specific stages where enrichment was observed.


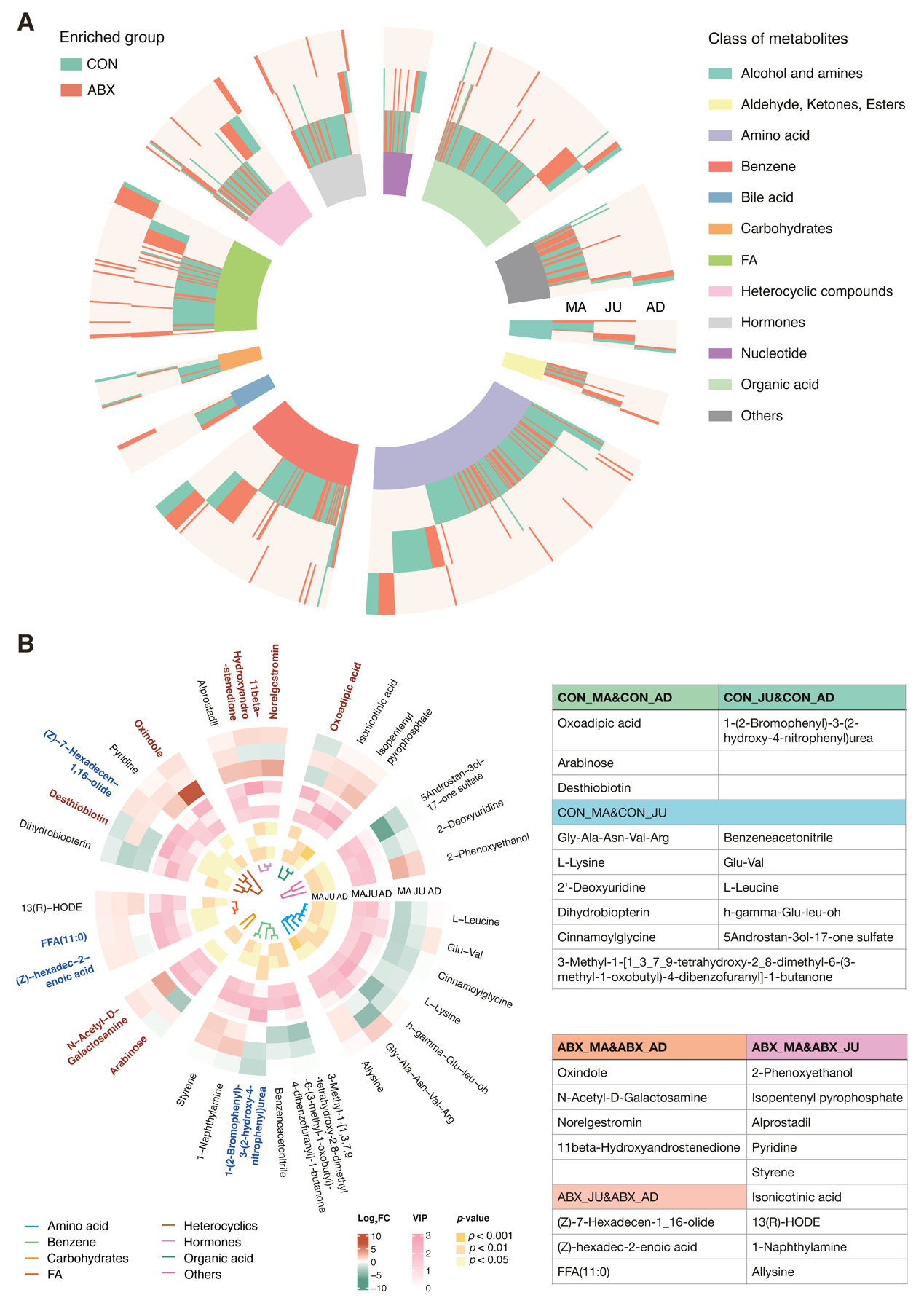


**Figure S8. Identification of consistent differential metabolites across developmental stages following maternal preconception antibiotic exposure.**

(A) Circular heatmap with metabolic class annotation displaying metabolites with significant abundance or prevalence differences between CON and ABX groups across the maternal (MA), juvenile (JU), and adult (AD) stages. (B) Multi-layered circular heatmap displaying conserved differential metabolites between CON and ABX groups across the maternal (MA), juvenile (JU), and adult (AD) stages. Conserved metabolites were defined as those showing consistent differential abundance (same direction) in at least two stages. The innermost ring represents the p-values of each metabolite, with darker colors indicating stronger statistical significance. The middle ring shows the VIP (variable importance in projection) scores, with darker shades denoting higher VIP values. The outermost ring illustrates the log₂ fold change (log₂FC), with red indicating enrichment in the ABX group and green in the CON group; greater color intensity reflects larger absolute log₂FC values. Table summarizing conserved differential metabolites, indicating the enriched group (CON or ABX) and the specific stages where differential abundance was observed.


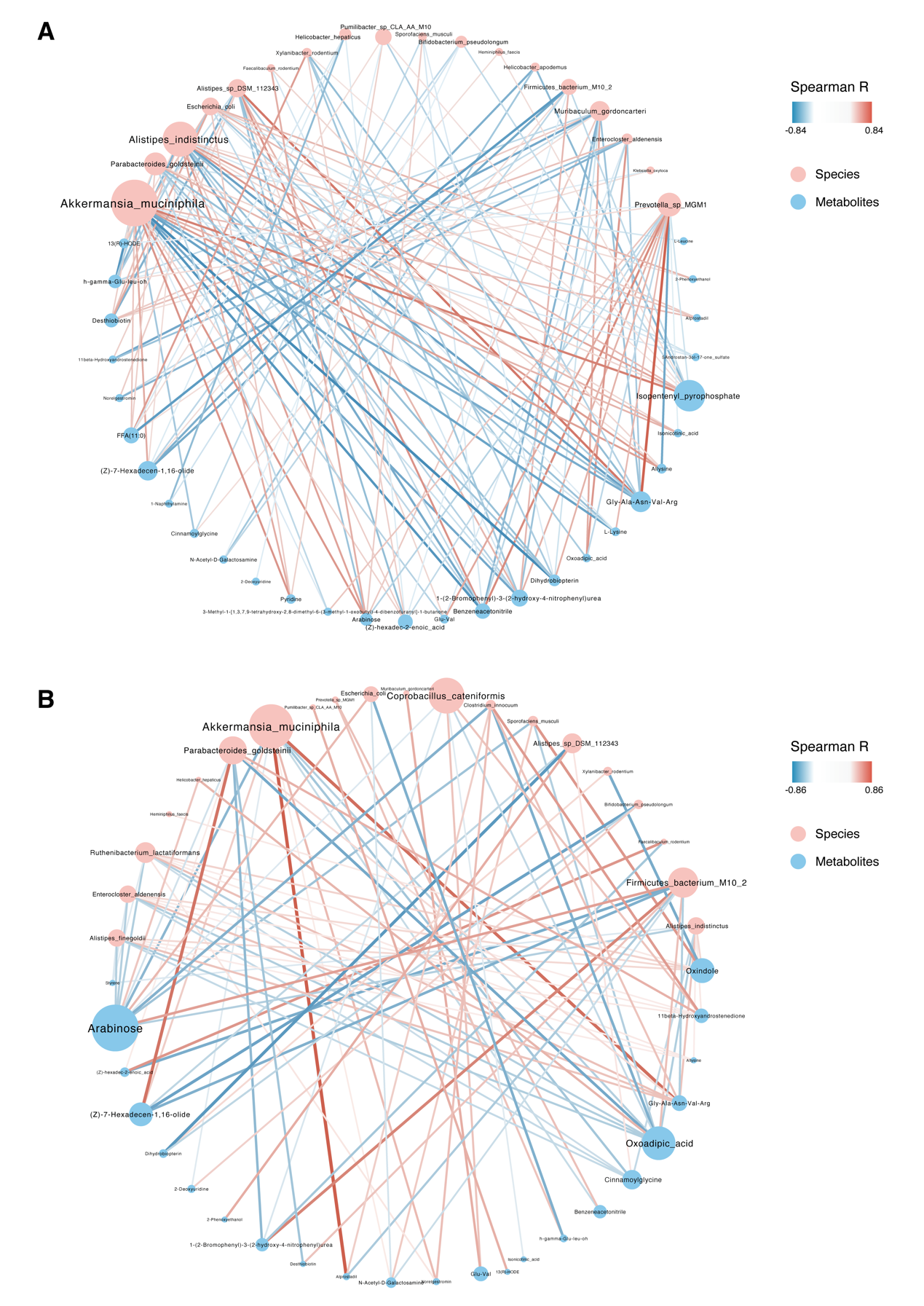


**Figure S9. Correlation networks between intergenerationally consistent differential species and metabolites in juvenile and adult offspring.**

(A) Spearman correlation network between conserved differential microbial species and metabolites in juvenile (JU) offspring. (B) Spearman correlation network in adult (AD) offspring. Edges represent significant correlations (p < 0.05, |R| > 0.5); red edges indicate positive correlations and blue edges indicate negative correlations. Greater correlation strength is reflected by darker edge colors and thicker lines. Orange nodes represent species, and blue nodes represent metabolites. Node size and label font size are proportional to betweenness centrality, indicating the importance of each feature in the network structure.


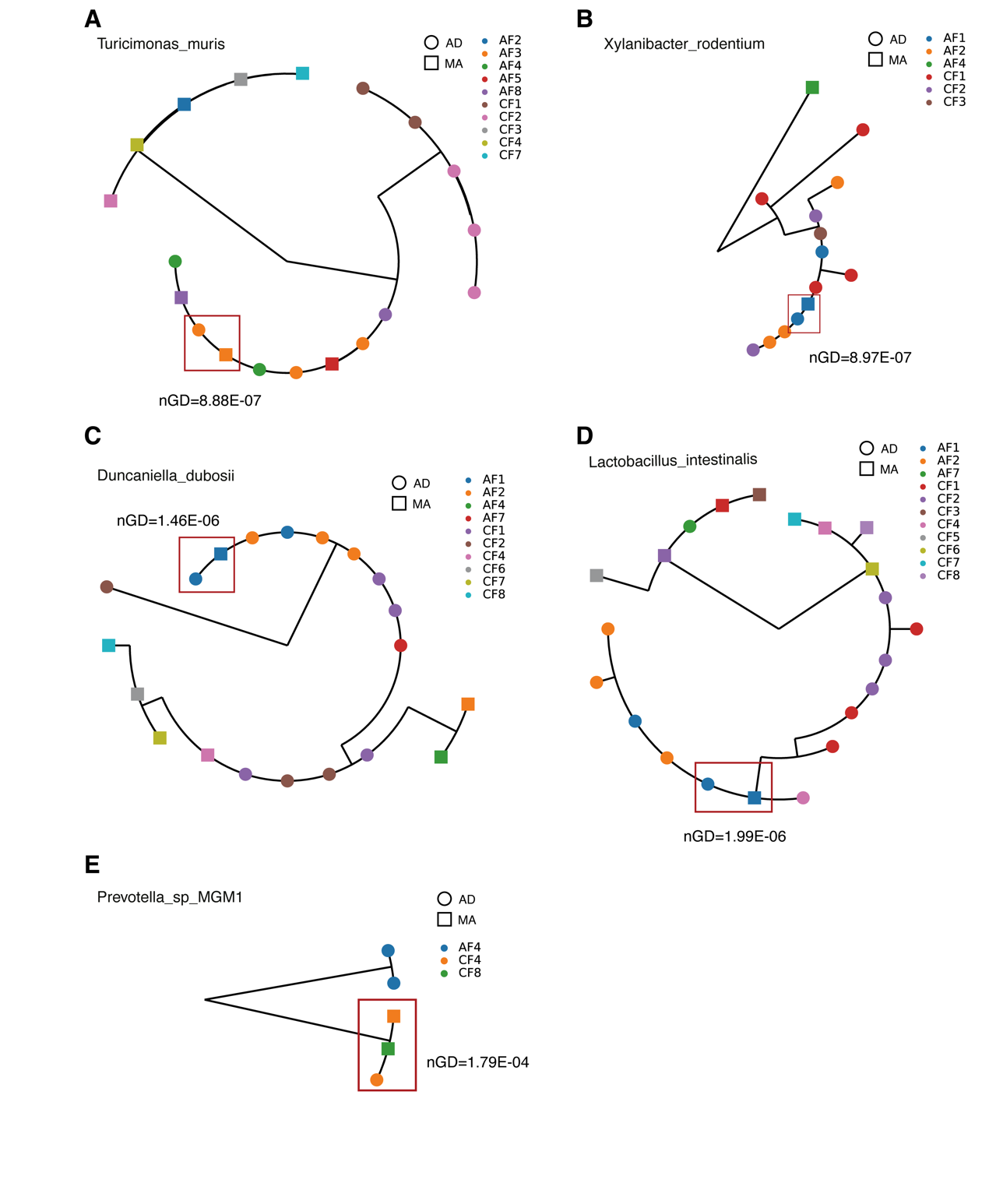


**Figure S10. Strain-sharing events identified by StrainPhlAn4 in preconception antibiotic exposure (ABX) and control (CON) groups.**

(A–D) Strain-sharing events in the ABX group for Turicimonas muris (A), Xylanibacter rodentium (B), Duncaniella dubosii (C), and Lactobacillus intestinalis (D). (E) Strain-sharing events in the CON group for Prevotella sp. MGM1. In all panels, circles represent adult offspring (AD) and squares represent maternal (MA). Different fill colors indicate distinct family groups. Calculated strain transmission events occurring between specific mother–child pairs are highlighted by red rectangles, and annotated with the their nGD.


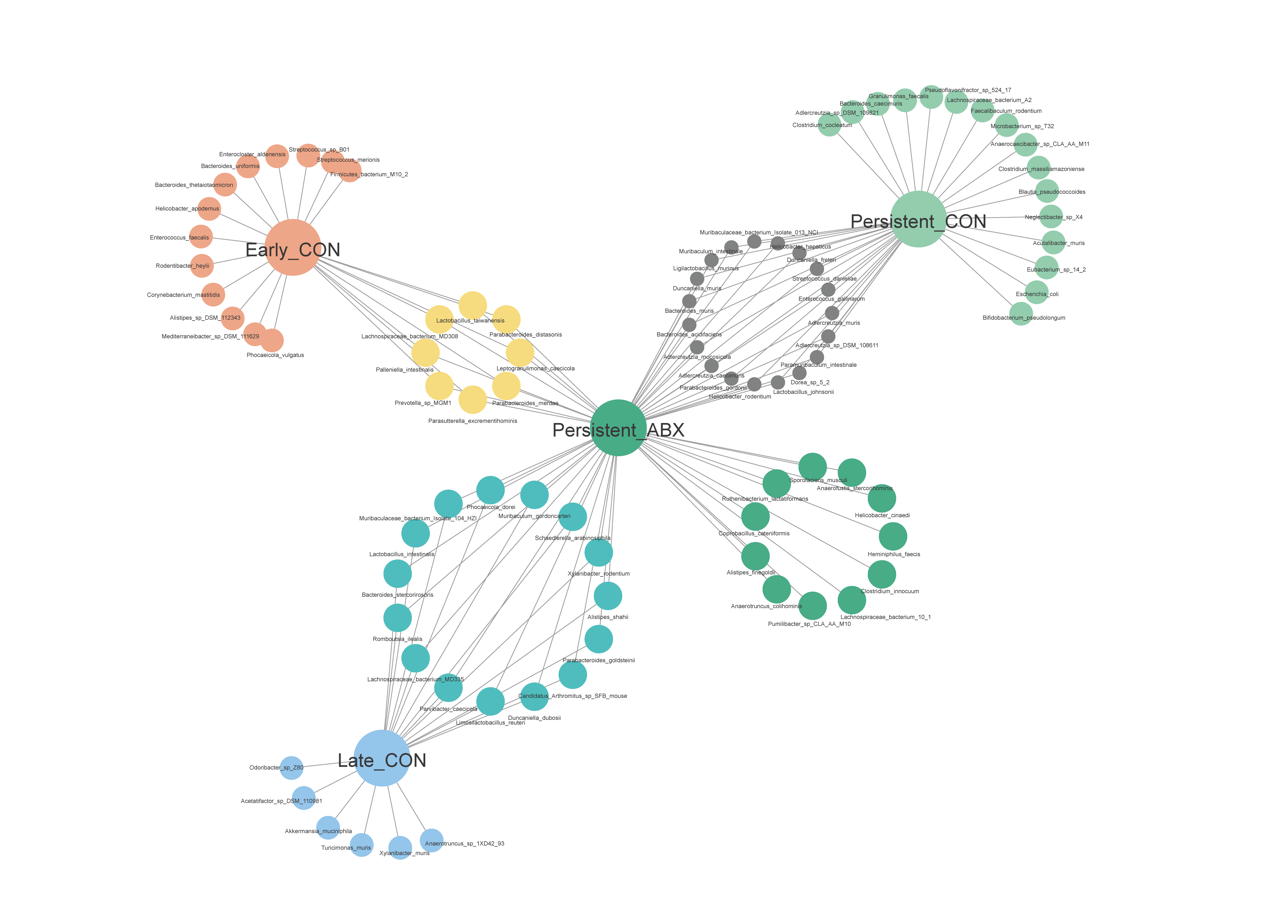


**Figure S11. Altered Colonization Patterns in Offspring Following Maternal Antibiotic Exposure.**

Colonization pattern comparison of ABX persistent species with colonization modes in the CON group. A network diagram highlights ABX-persistent species (Persistent_ABX) and their overlap with species in three colonization categories in the CON group: early (more prevalent at JU; Early_CON), persistent (similar prevalence in JU and AD; Persistent_CON), and late (more prevalent at AD; Late_CON) colonization. Lines represent shared species between ABX-persistent species and each colonization category in the CON group. Species not overlapping with any category are shown as only linked with Persistent_ABX.


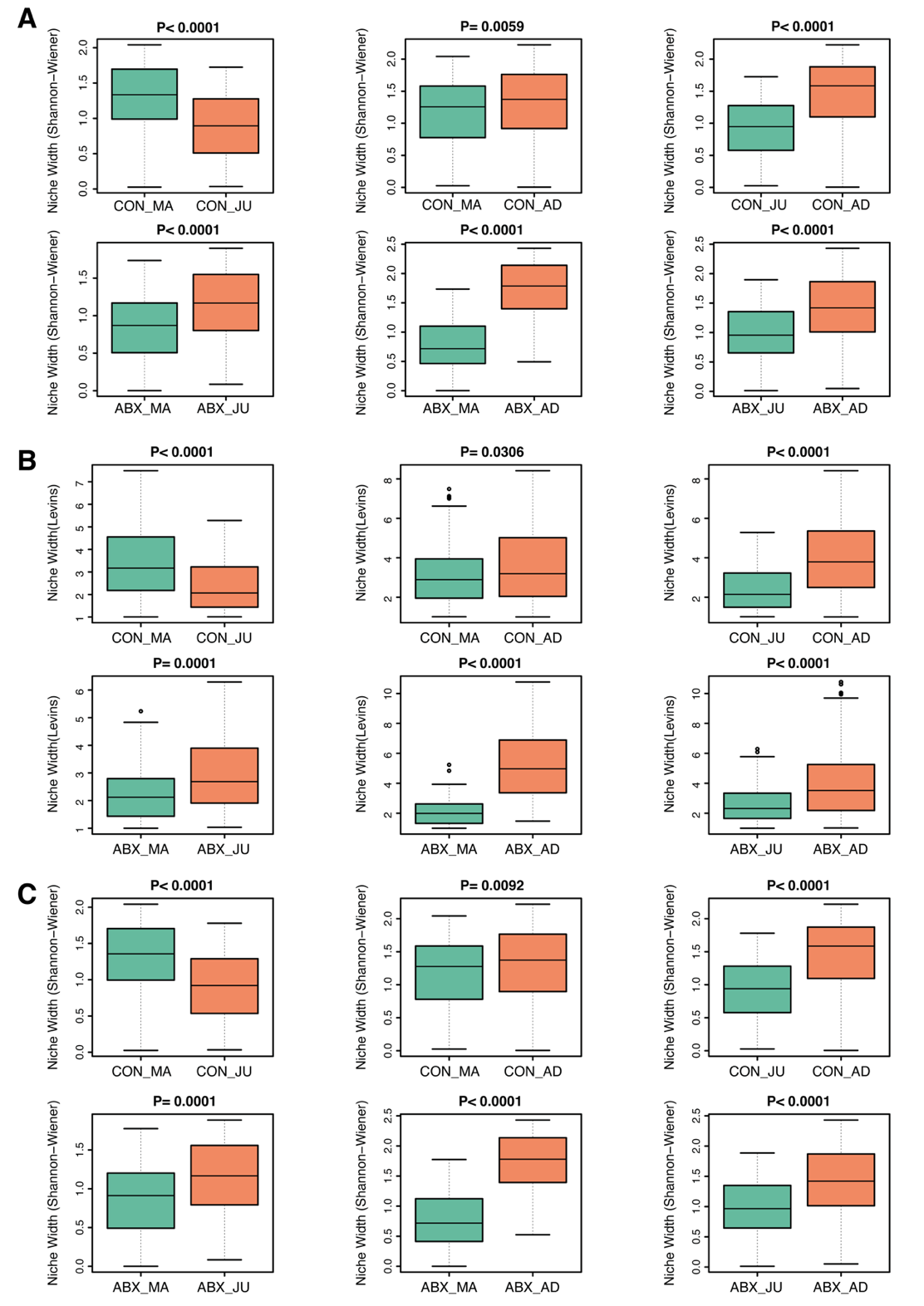


**Figure S12. Niche breadth of shared species between developmental stage pairs.**

(A) Niche breadth calculated using the Shannon-Wiener index. Three stage comparisons are shown: MA and JU, MA and AD, and JU and AD. (B) Niche breadth calculated using Levins index, accounting for detection limits by rarefying each sample to the minimum sequencing depth before calculation. (C) Niche breadth calculated using the Shannon-Wiener index, after rarefying to the minimum sequencing depth (analogous to panel B). P-values were assessed by the Wilcoxon rank-sum test and are reported to four decimal places.


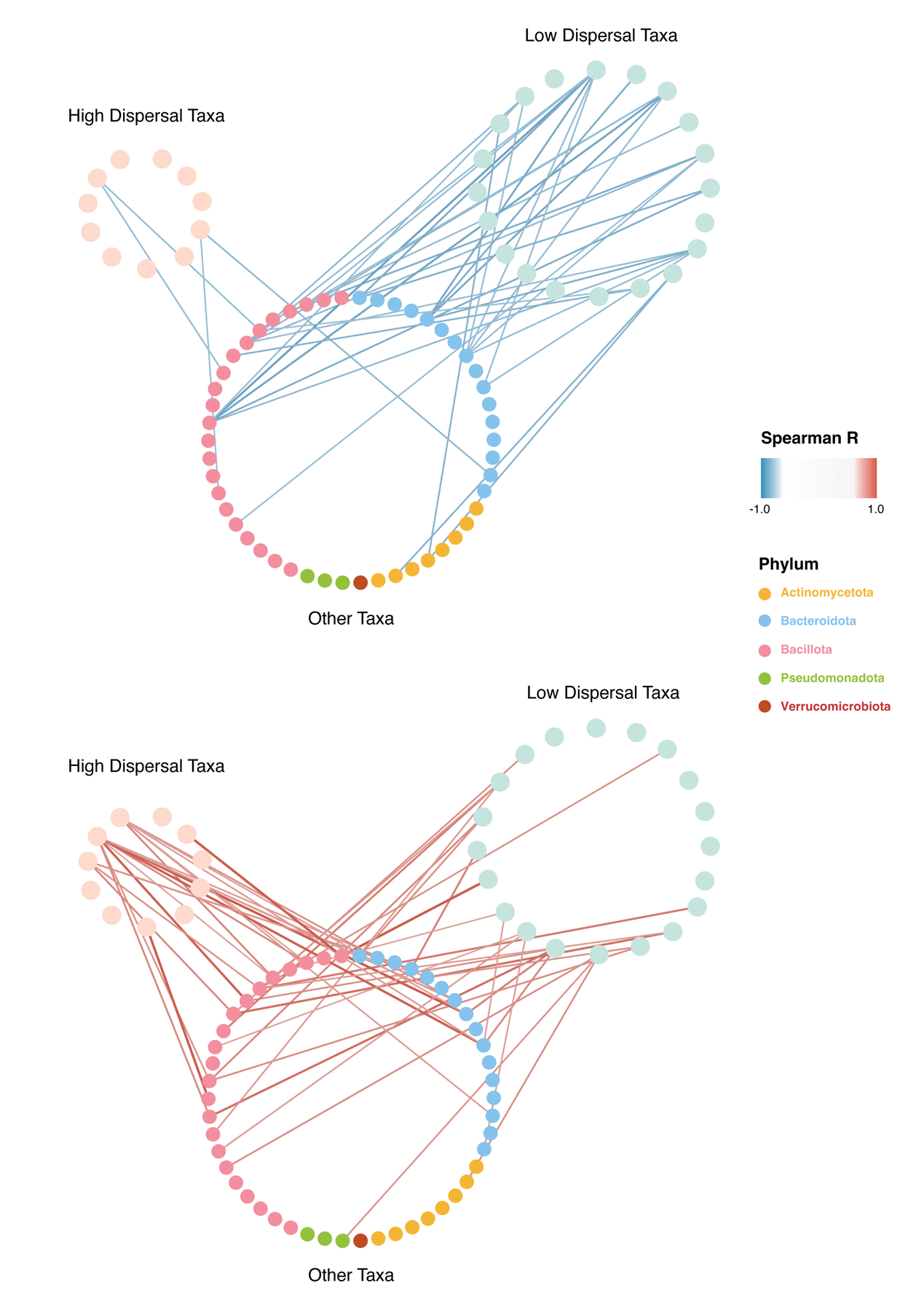


**Figure S13. Co-occurrence network of taxa with different dispersal capacities in ABX-AD.**

Taxa were previously classified into three categories based on their dispersal ability in ABX-AD compared to CON-AD: High Dispersal Taxa (orange nodes), Low Dispersal Taxa (green nodes), and Other Taxa (center nodes, colored with phylum information). Within ABX-AD samples, Spearman correlation networks were constructed to assess interactions between high/low dispersal taxa and other taxa. Edges represent strongly significant correlations (|r| > 0.8, p < 0.05); red edges denote positive correlations and blue edges indicate negative correlations. The upper panel highlights negative correlations, where low-dispersal taxa tend to exhibit broad negative associations with other taxa. The lower panel highlights positive correlations, with high-dispersal taxa displaying preferential positive interactions with other community members.


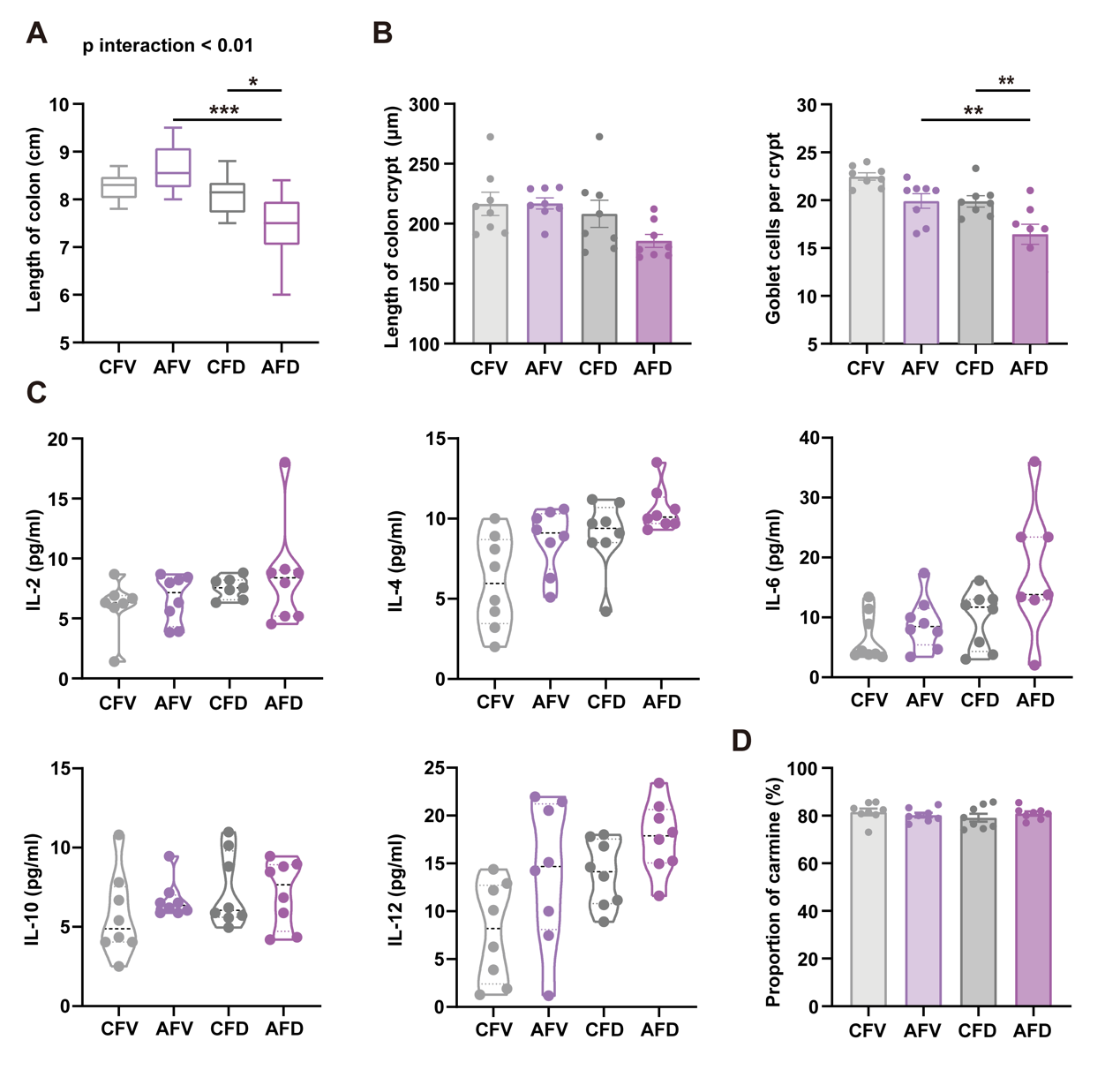


**Figure S14. Effects of DSS on colon length, intestinal development, inflammatory factors, and small intestine transit in mice receive FMT form adult offspring of ABX dams.**

(A) Length of the colon (n=8). (B) Crypt depth in the colon and goblet cells in the colonic crypt of adult offspring using H&E or Alcian Blue-Periodic Acid Schiff (AB-PAS) staining (n = 8). (C) Concentrations of inflammatory factors (IL-2, IL-4, IL-6, IL-10, IL-12) (n = 8). (D) Small intestine transit time (n = 8). Data are presented as mean ± SEM. Two-way ANOVA followed by Tukey’s multiple comparison test was used for analyses involving two variables (A, B, C, D). **P* < 0.05, ***P* < 0.01, ****P* < 0.001.

**
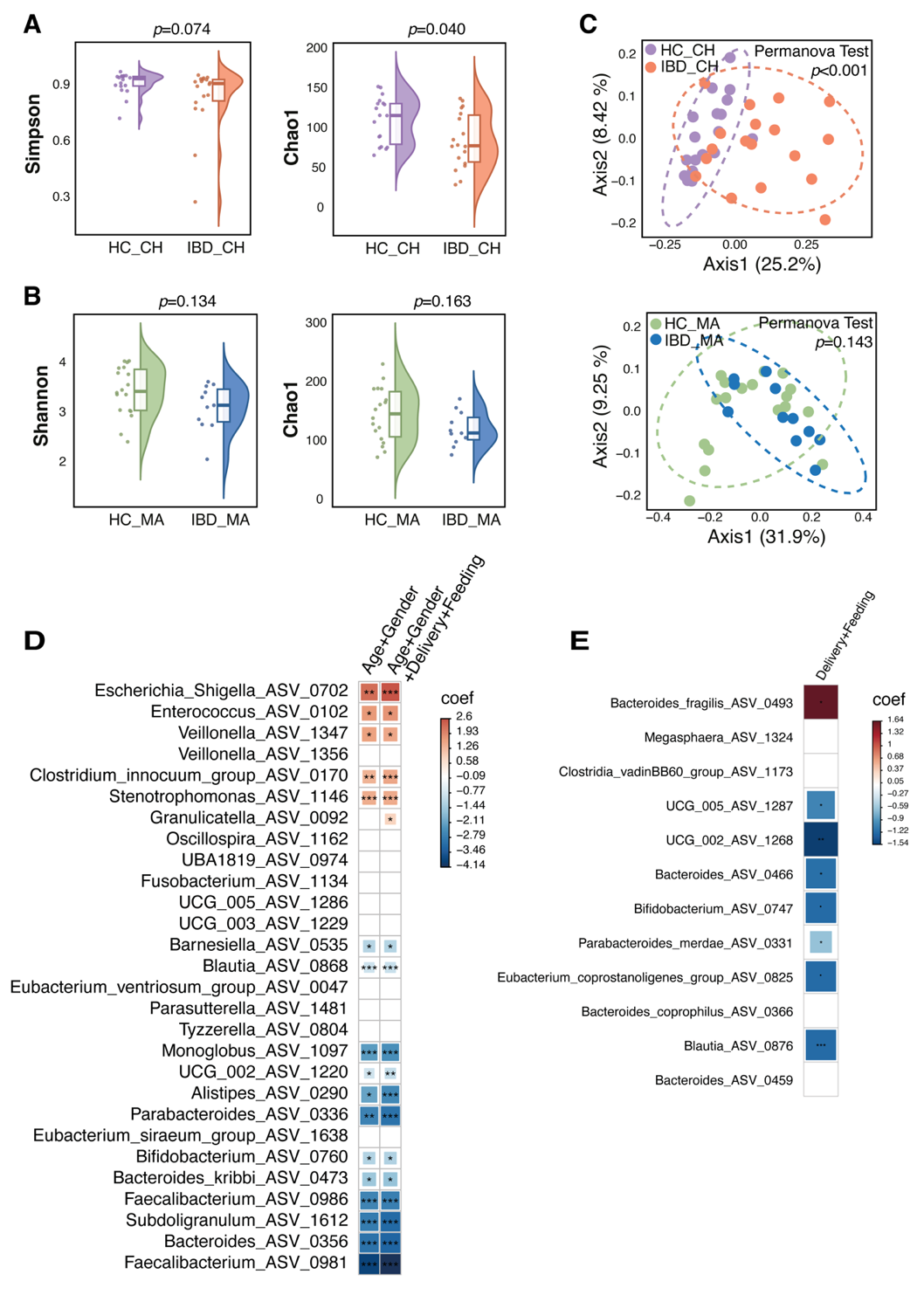
**

**Figure S15. Gut microbiome diversity and differential taxa adjusted for covariates in children and mothers.**

(A) Simpson and Chao1 indices in children (IBD-CH vs. HC-CH, n=18). (B) Shannon and Chao1 indices in mothers (IBD-MA vs. HC-MA, n=11,18). (C) PCoA based on Bray–Curtis distances for children and mothers. (D-E) Heatmap of differentially abundant taxa in children(D) and mothers(E). LEfSe-identified taxa are colored by coefficients from covariate-adjusted MaAsLin2 models; only cells with p < 0.05 are filled. Alpha diversity statistical significance was assessed by the Wilcoxon rank-sum test. Significance level: * *p* < 0.05, ** *p* < 0.01, *** *p* < 0.001.


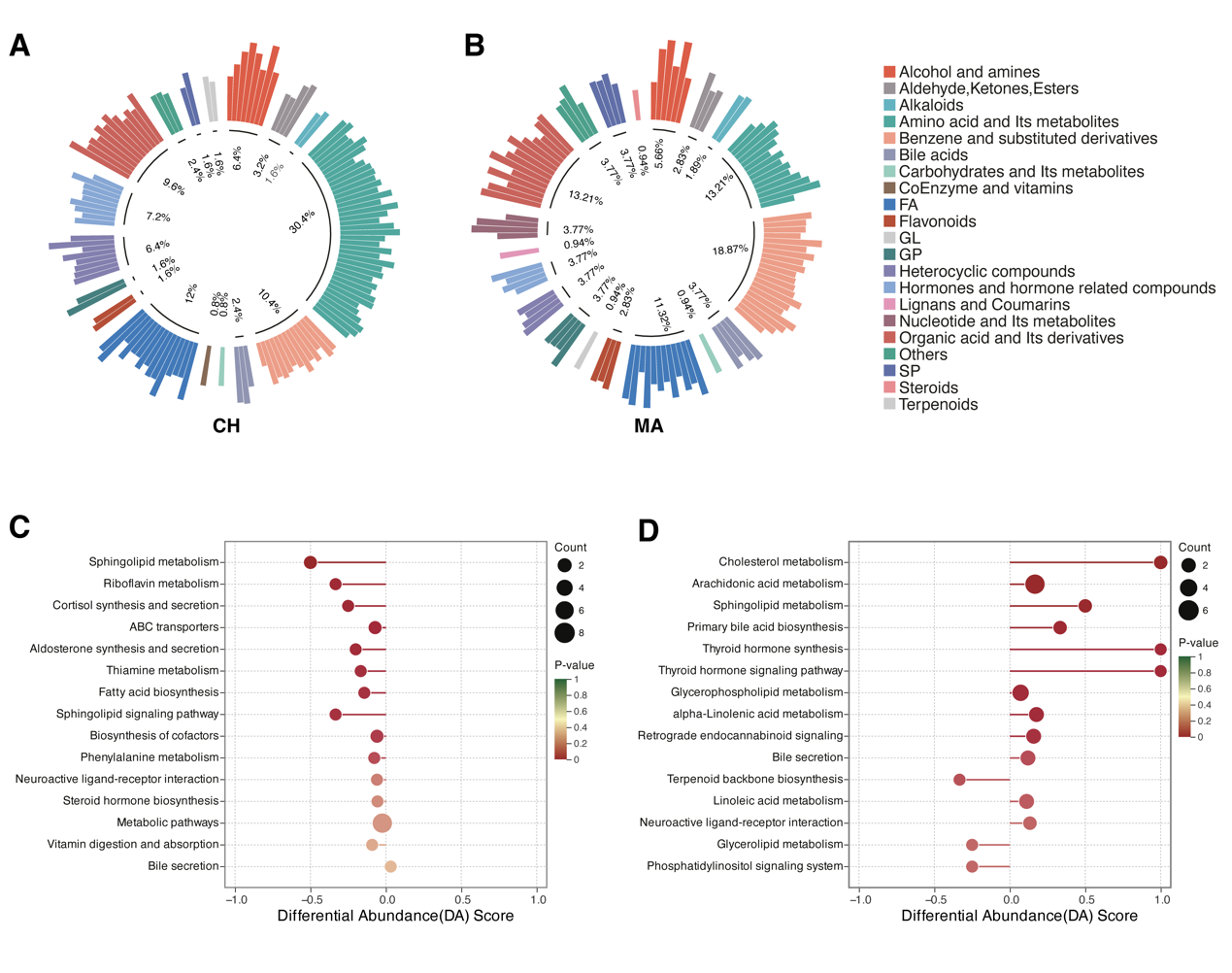


**Figure S16. Differential metabolites in children and mothers.**

(A) Proportional distribution of primary categories of differential metabolites in children. (B) Proportional distribution of primary categories of differential metabolites in mothers. (C) KEGG pathway enrichment of differential metabolites displayed as lollipop plots for children. (D) KEGG pathway enrichment of differential metabolites displayed as lollipop plots for mothers. Lollipop length indicates the Differential Abundance (DA) score; dot size represents the number of metabolites enriched in the pathway; color scale (redder = more significant) reflects p-value.


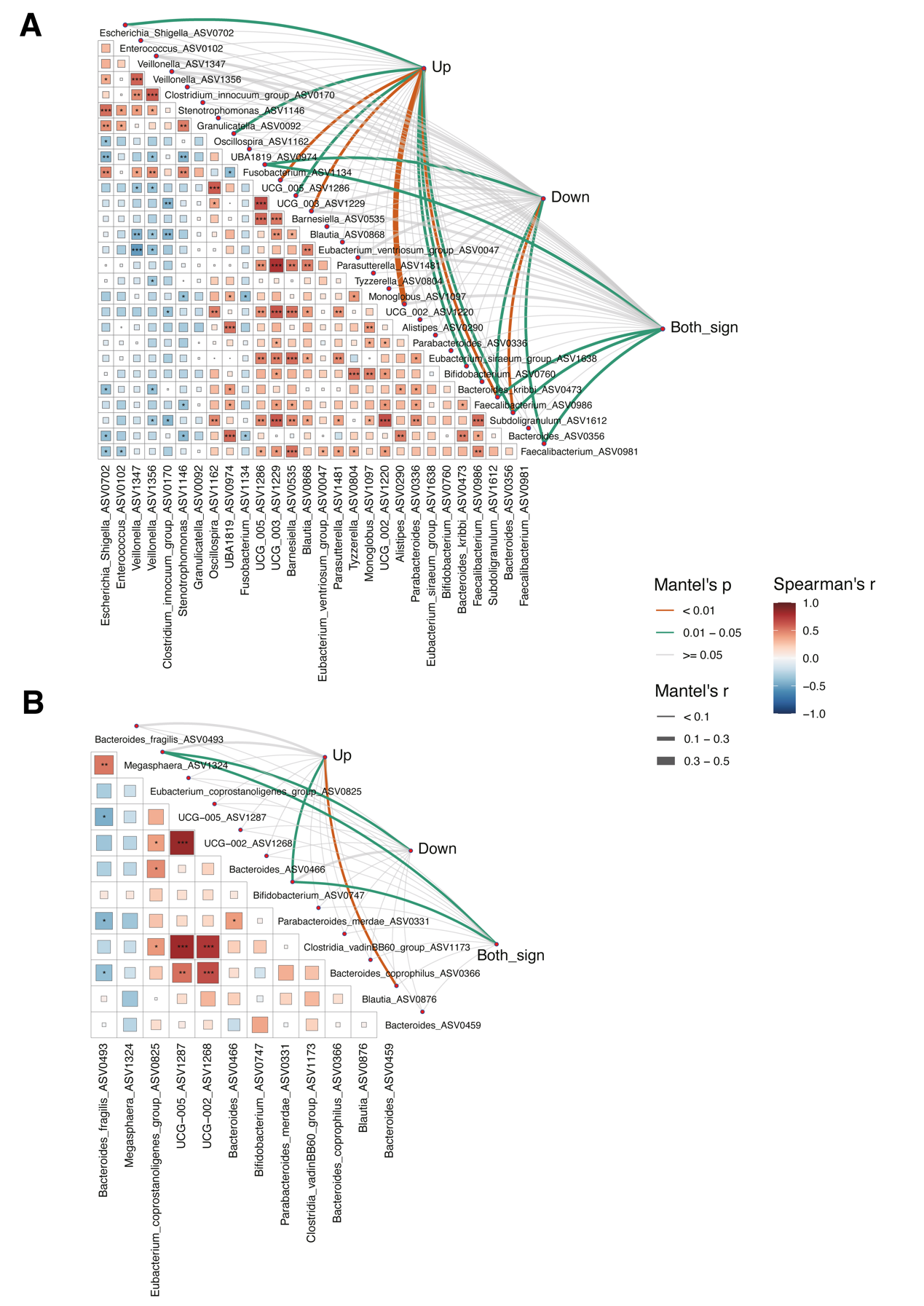


**Figure S17. Microbe-metabolite associations in children and mothers.**

(A) Mantel test correlation analysis between differential microbial ASV and differential metabolites in children group. (B) Mantel test correlation analysis between differential microbial ASV and differential metabolites in mothers group. The heatmaps show pairwise Spearman correlations between differential ASVs and metabolites; red and blue shading indicate positive and negative correlations, respectively, with intensity proportional to |r| value. Statistical significance level: *p < 0.05, **p < 0.01, ***p < 0.001. Mantel test results are visualized by line thickness indicating the Mantel r value, and line color representing significance: green lines for 0.01 ≤ p < 0.05, and red lines for p < 0.01.


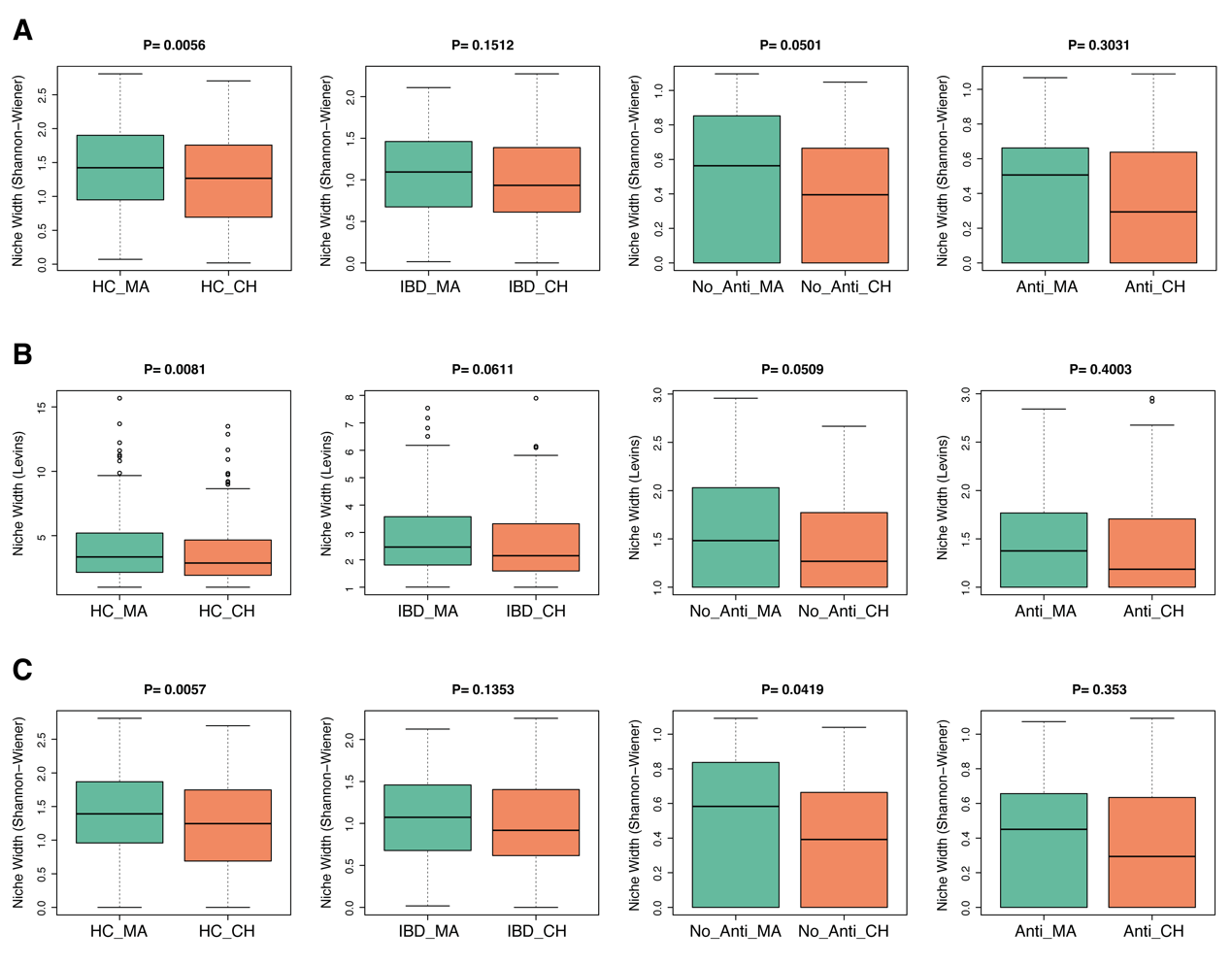


**Figure S18. Comparison of maternal and child microbial niche breadth under different stratifications.**

(A) Niche breadth calculated using Shannon-Wiener index in different mother–child groups. (B) Niche breadth calculated using Levins index, accounting for detection limits by rarefying to the minimum sample sequencing depth before calculation. (C) Niche breadth calculated using Shannon-Wiener index, after rarefying to the minimum sample sequencing depth. P-values were assessed by the Wilcoxon rank-sum test and are reported to four decimal places.
